# Monsoon intensification in East Asia triggered the evolution of its flora

**DOI:** 10.3389/fpls.2022.1046538

**Published:** 2022-11-25

**Authors:** Jun-Wei Ye, Bin Tian, De-Zhu Li

**Affiliations:** ^1^ Key Laboratory for Forest Resources Conservation and Utilization in the Southwest Mountains of China, Ministry of Education, Southwest Forestry University, Kunming, China; ^2^ Germplasm Bank of Wild Species in Southwest China, Kunming Institute of Botany, Chinese Academy of Sciences, Kunming, China

**Keywords:** east asian monsoon, endemism, late miocene, pleistocene, tertiary relict

## Abstract

**Introduction:**

East Asia (EA), which falls within the region of the Asian monsoon that is composed of the East Asia monsoon (EAM) and the Indian monsoon (IM), is known for its high species diversity and endemism. This has been attributed to extreme physiographical heterogeneity in conjunction with climate and sea-level changes during the Pleistocene, this hypothesis has been widely proven by phylogeographic studies. Recently, dated phylogenies have indicated that the origins (stem age) of the flora occurred after the Oligocene–Miocene boundary and are related to the establishment of the EAM.

**Methods:**

Hence, this study further examined whether the strengthening of the monsoons triggered floral evolution via a meta-analysis of the tempo-spatial pattern of evolutionary radiation dates (crown ages) of 101 endemic seed plant genera.

**Results:**

Taxonomic diversification began during the late Eocene, whereas the accumulated number of diversifications did not significantly accelerate until the late Miocene. The distribution of the weighted mean and the average divergence times in the EAM, IM, or transitional regions all fall within the mid-late Miocene. Fossils of the Tertiary relict genera are mostly and widely distributed outside EA and only half of the earliest fossils in the EA region are not older than Miocene, while their divergence times are mostly after the late Miocene. The pattern of divergence time of monotypic and polytypic taxa suggest the climatic changes after the late Pliocene exert more influence on monotypic taxa.

**Discussion:**

The two key stages of floral evolution coincide with the intensifications of the EAM and IM, especially the summer monsoon which brings a humid climate. An integrated review of previous studies concerning flora, genus, and species levels further supports our suggestion that monsoon intensification in EA triggered the evolution of its flora.

## Introduction

**Figure 1:**
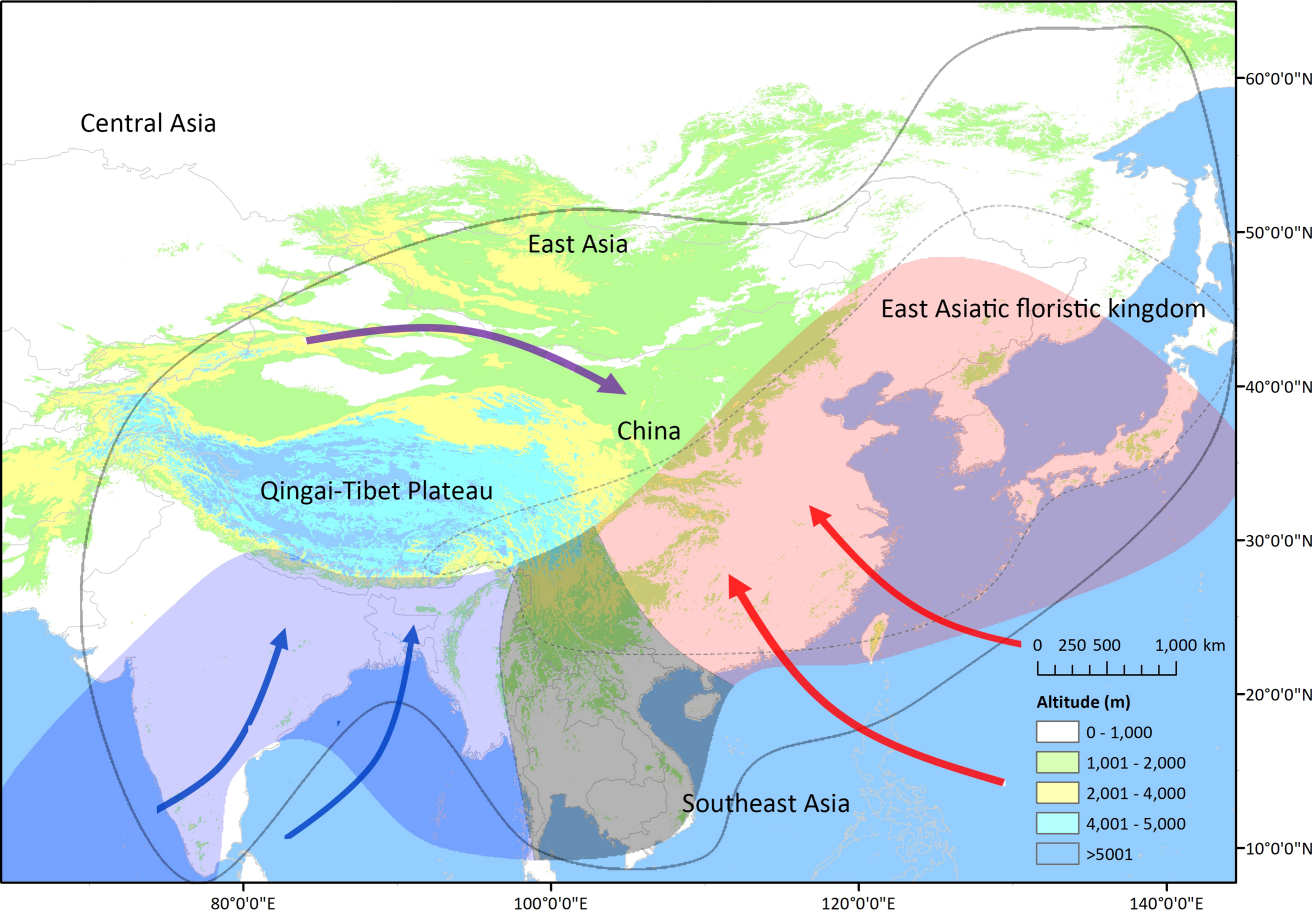
The East Asian monsoon system which encompasses the East Asian summer monsoon (EASM, red arrows), the Indian summer monsoon (ISM, blue arrows), and the Asian winter monsoon (AWM, purple arrows). Ranges of East Asia (Manchester et al., 2009) and East Asiatic floristic kingdom (Wu and Wu, 1996) are illustrated through a black line and a dotted line. The modern summer monsoon areas are those recognized by Wang and Ho (2002); red, blue, and gray areas indicate the EASM, ISM, and transitional region of the EASM and ISM, respectively.

East Asia (EA, **Figure 1**) is characterized by high levels of plant diversity and endemism (Qian, 2002; Manchester et al., 2009). It harbors 75% of extant gymnosperm families (Christenhusz et al., 2011) and more than 60% of angiosperm families (Apgiv, 2016). Moreover, it has higher diversity at the genus and species levels than those in other regions of the Northern Hemisphere (e.g., 1.5 times that of North America) (Qian, 2002). In addition, there are approximately 600 endemic seed plant genera, including many relicts, notably *Ginkgo*, *Metasequoia*, *Glyptostrobus*, *Taiwania*, *Liriodendron*, and *Davidia*, which have very ancient origins (mostly >100 million years ago, Ma) (Manchester et al., 2009). Thus, the evolutionary patterns and mechanisms of flora in EA have attracted much attention and have been continuously investigated.

Wu and Wu (1996) proposed that the former “Eastern Asiatic region” is composed of an independent “East Asiatic floristic kingdom” (**Figure 1**) or “Sino-Japanese floristic region” and is likely to be the origin or center of diversification for extant angiosperms in the Northern Hemisphere. Extreme physiographical heterogeneity, periodic glacial–interglacial climatic alternations, and accompanying sea-level fluctuations (rise in interglacial periods and fall in glacial periods) during the Pleistocene provided abundant opportunities for evolutionary radiation, resulting in extremely high levels of plant diversity in EA (Qian and Ricklefs, 2000; Harrison et al., 2001). Subsequent phylogeographic studies have revealed the complicated influence of changes in climate (e.g., global cooling, glacial–interglacial alternations), topography (e.g., mountain uplift, river drainage pattern change), and/or sea level (rise or fall) on the current genetic structure of plants in EA (Qiu et al., 2011; Ye et al., 2017a). However, the evolution of some plants can be attributed to geoclimatic changes prior to the Pleistocene, such as the uplift of the Tibetan Plateau (TP), global cooling, and monsoon development (Favre et al., 2015; Ye et al., 2017a).

The EA region is under the effect of the Asian Monsoon system which is composed of the East Asia monsoon (EAM) and the Indian monsoon (IM) (Wang and Ho, 2002). The ancient pattern of the Asian Monsoon has expanded northward to the subtropical region during Eocene, probably driven by the uplift of the TP, global cooling, and rapid regression of the proto-Paratethys Sea (Licht et al., 2014; Wu et al., 2022). The modern-like pattern of the EAM appeared between the late Oligocene and early Miocene, caused also by the TP uplift and global cooling, and other historical changes, such as high-latitude temperature changes (Lu and Guo, 2014; Clift et al., 2015; Deng et al., 2021). While, the modern-like IM is likely to have arisen in response to the presence of a high (>5 km) Himalaya after the mid-Miocene (Spicer, 2017).

As there was a vast arid zone from southeast to northwest before the Neogene and the arid belt also exerted a great influence on the genetic divergence of endemic plants during the late Miocene or late Pliocene (Guo et al., 2008; Bai et al., 2016; Ye et al., 2017b), and precipitation is a key factor controlling the plants’ growth, particularly the phenology, and the distribution of vegetation in the subtropical region of EA (~20°N to 35°N) (Li et al., 2021), the summer monsoons, the East Asia summer monsoon (EASM), and the Indian summer monsoon (ISM) (**Figure 1**), which bring humid climate, are proposed to be critical to the floral evolution of EA (Wu et al., 2010). The EASM experienced two major monsoon intensifications that occurred during the late Miocene and late Pliocene (An et al., 2001; Wan et al., 2006; Clift et al., 2015); intensifications during the mid-Miocene (Farnsworth et al., 2019) and the Pleistocene (An et al., 1990) have also been reported. The strengthening of the ISM during the middle–late Miocene (Spicer, 2017) and a gradual intensification during the late Pliocene (Chang et al., 2010) are suggested.

Recent studies on floral scales indicate that their origin in EA was likely triggered by monsoon development. The origin of over 200 clades of seed plants in the Sino-Japanese floristic region indicates that most clades have originated (stem age) since the early Miocene (22.23 Ma); moreover, the two subkingdoms are probably of a similar age (Chen et al., 2018). In dated phylogeny of the angiosperm genera in China, 66% genera did not originate until early in the Miocene (23 Ma) (Lu et al., 2018). Additionally, biogeographic studies indicate that many characteristic taxa in subtropical evergreen broadleaved forests (EBLFs) also originated during the late Oligocene or early Miocene, such as *Schima*, *Castanopsis*, *Machilus*, *Quercus* section Cyclobalanopsis, and *Dendrobium* (Xiang et al., 2016; Deng et al., 2017; Yu et al., 2017). Most recently, Hai et al. (2022) also have shown that modern EBLFs did not begin to appear until Miocene (23 Ma) through dated phylogeny of dominant Fagaceae species. This indicates that floral taxa have originated in EA due to the EAM, especially the EASM, which caused a humid climate between the late Oligocene and early Miocene (Clift et al., 2015). Accelerated diversifications linked to intensifications during the late Miocene and late Pliocene are also reported (Yu et al., 2017; Ye and Li, 2022).

A meta-analysis has been used previously to investigate the floral evolution in the Sino-Japanese floristic region (Chen et al., 2018) or Hengduan Mountains (Xing and Ree, 2017). Recently, Ye and Li (2022) have explored the diversifications of subtropical EBLFs over the last eight million years through a compilation of origin times of dominant species; Li et al. (2022) have investigated the timing and mode of cave colonization through a multi-taxon meta-analysis combined with the investigation of paleoenvironmental dynamics. So, in the present study, we further tested whether the strengthening of the Asian monsoon has triggered the floral evolution in EA *via* meta-analyses of the tempo-spatial pattern of radiation dates (crown ages) of endemic seed plant genera.

## Methods

### Diversification time and fossil data

The diversification times (crown ages) of the endemic genera were obtained through dated phylogeny in polytypic genera or phylogeography (coalescence time of haplotypes across distributions) in monotypic genera (**Supplementary Table S1**). The median and 95% highest-probability density interval of the divergence time for the endemic seed plant genera were collected. The mean and median values of the diversification times were calculated. The number and accumulation of genera diversified in every five million years were calculated and graphed. Diversification times between monotypic and polytypic genera was compared. The first appearance of fossils of endemic relict genera outside or inside of EA were compiled mainly from the studies of Manchester et al. (2009), Chen et al. (2018), and Tang et al. (2018).

### Spatial distribution of diversification time

To illustrate the spatial distribution of diversification times, we divide EA into different regions. Because it is hard to collect distribution data based on biogeographical regions, level-3 regions defined in the Taxonomic Databases for Plant Sciences (TDWG, Brummitt, 2001) based on botanical countries or aggregations of constituent provinces in a large country fit well to the monsoon climate regions, and the Plants of the World Online (POWO, https://powo.science.kew.org/) database provides well-collected distribution data according to TDWG codes, so 29 regions, namely, Amur, Assam, Bangladesh, Cambodia, China North-Central, China South-Central, China Southeast, East Himalaya, West Himalaya, Hainan, India, Inner Mongolia, Japan, Khabarovsk, Korea, Laos, Manchuria, Mongolia, Myanmar, Nansei-shoto, Nepal, Primorye, Qinghai, Sakhalin, Taiwan, Thailand, Tibet, Vietnam, and Xinjiang (Brummitt, 2001), were identified. The weighted mean diversification times (MDTs, Lu et al., 2018) and average divergence times (ADTs) in each region were calculated by integrating spatial distribution and radiation dates. *AGEi* represents the diversification times of a genus *i* (*i* = 1, …, *n*) in a region, and *Si* is the species number in the genus. From this, MDT and ADT were calculated as:


$$MDT=\frac{\left(AGE1\times S1\right)+\left(AGE2\times S2\right)+\left(AGE3\times S3\right)+\ldots +\left(AGEn\times Sn\right)}{S1+S2+S3+\ldots +Sn}$$

$$ADT=\frac{AGE1+AGE2+AGE3+AGEn}{n}$$

Spatial distribution and species number data of all genera were obtained from the POWO database.

## Results

### Temporal mode

Overall, we obtained divergence times for 96 taxa (101 genera), including seven complexes (*Ombrocharis/Perillula, Soroseris/Stebbinsia, Oresitrophe/Mukdenia, Changium/Chuanminshen, Physochlaina/Przewalski, Perilla/Keiskea, and Akebia/Archakebia*) and four lineages (*Phtheirospermum*-1 and *Phtheirospermum*-2 and *Syncalathium*-1 and *Syncalathium*-2), and the taxa comprised 26 trees, 20 shrubs, 48 herbaceous plants, and 3 lianas in 76 polytypic and 20 monotypic taxa (**Supplementary Table S1**).

**Figure 2:**
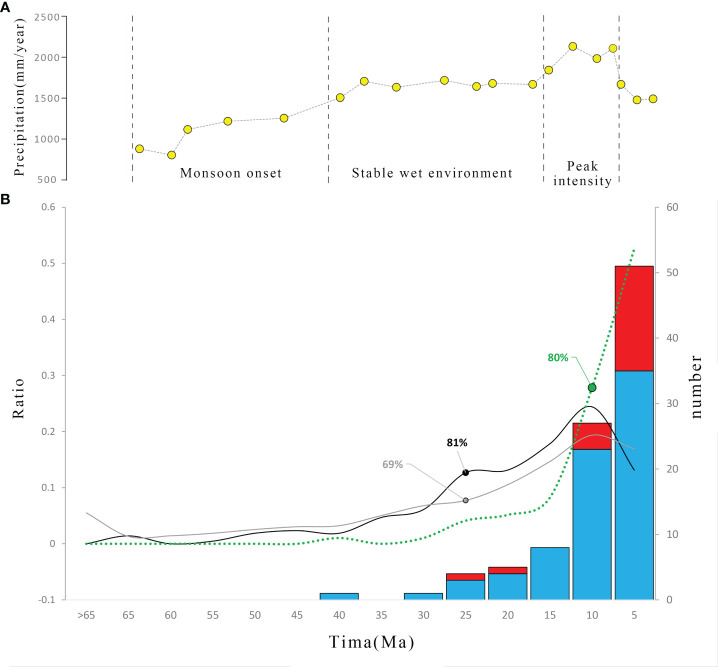
**(A)** Monsoon conditions are indicated by the modeled mean annual precipitation for each geologic stage, denoted by dotted black lines at idealized CO^2^ levels (yellow circles) modified through Farnsworth et al. (2019). **(B)** The number and ratio (dotted green) of diversifications of endemic seed taxa in East Asia in five-million-year increments. Blue and red represent polytypic and monotypic taxa, respectively. Black and gray lines indicate the ratio of genera/clades origins during each increment in the East Asiatic floristic kingdom (Chen et al., 2018) or Chinese angiosperms (Lu et al., 2018), respectively. Accumulations at the Oligocene–Miocene boundary (25 Ma) in the East Asiatic floristic kingdom (81%) or Chinese angiosperms (69%) and late Miocene (10Ma, 80%) in endemic seed plant genera are labeled.

The oldest divergence was dated to the late Eocene (35.91 Ma, *Hovenia*) and the youngest to the late Pleistocene (0.04 Ma, *Gaoligongshania*). The mean divergence time was 6.94 Ma with a 95% confidential interval (CI) of 5.54–8.34 Ma (median = 4.52 Ma). Diversification events slowly accumulated before a bloom after the late Miocene, and 80% (*n* = 77) of the sampled genera diversified after 10 Ma (**Figure 2**). However, radiations of monotypic taxa were more recent (median = 4.05 Ma) than those of polytypic taxa (median = 7.84 Ma) (**Supplementary Figure S1**). Among monotypic taxa, 80% (16/20) had population divergence after the Miocene (<5.3 Ma) and 55% (11/20) diverged during the Pleistocene (**Figure 2** and **Supplementary Table S1**). Accelerated accumulation of radiation events after the late Miocene (77%, 59/77) also occurred in the polytypic taxa (**Supplementary Figure S2**).

We found that 23 of 101 sampled genera are represented in fossils (**Table 1**). The earliest fossils known are found mostly (22/23) before the Miocene with the oldest dating to Triassic (*Ginkgo*), while the relictualism is mainly (83%, 19/23) diverged after late Miocene (**Supplementary Table S1**), and about half (12/23) of the earliest fossils in the EA region are not older than Miocene with ancient genera that could be traced back to Cretaceous or even the Jurassic period (**Table 1**).

**Table 1:** Earliest fossils of the East Asian endemic Tertiary relict genera.

Genus	Family	Date of earliest known fossil	Country where the earliest fossil was found	Location/oldest fossil inside of EA	References
*Ginkgo*	Ginkgoaceae	Triassic	**CN**, RU, AU, GE, UZ, UA, CA	China/Triassic	Manchester et al. (2009), Chen et al. (2018), Tang et al. (2018)
*Cryptomeria*	Cupressaceae	Paleocene	IE	Japan/Miocene	
*Glyptostrobus*	Taxodiaceae	Cretaceous	**CN, JP**, US, CA	China, Japan/Cretaceous	
*Pterostyrax*	Styracaceae	Oligocene	DE	Japan/middle Pleistocene	
*Tapiscia*	Tapisciaceae	Eocene	US, DE	China/Miocene	
*Keteleeria*	Pinaceae	Eocene	CA	China/Miocene	
*Koelreuteria*	Sapindaceae	Cretaceous	CA	–	
*Taiwania*	Cupressaceae	Cretaceous	US, CA, RU	Japan/early Miocene	
*Akebia*	Lardizabalaceae	Miocene	**JP**, DE	Japan/Miocene	
*Davidia*	Davidiaceae	Cretaceous	CA	Japan/late Miocene	
*Emmenopterys*	Rubiaceae	Eocene	US, DE	–	
*Cunninghamia*	Taxodiaceae	Early Cretaceous	**CN, JP**, US, CA	China, Japan/early Cretaceous	
*Euptelea*	Eupteleaceae	Eocene	US	China/middle Miocene	
*Trochodendron*	Trochodendraceae	Cretaceous	**JP**	Japan/Cretaceous	
*Amentotaxus*	Taxaceae	Cretaceous	CA	–	
*Sargentodoxa*	Lardizabalaceae	Eocene	US, DE	–	
*Tripterygium*	Celastraceae	Late Miocene	**JP**	Japan/late Miocene	
*Tetracentron*	Trochodendraceae	Paleocene	**CN**, JP, RU	Japan/middle Miocene	
*Dipelta*	Caprifoliaceae	Eocene	US, UK	–	
*Dipteronia*	Aceraceae	Paleocene	US, RU	China/early Oligocene^a^	
*Cyclocarya*	Juglandaceae	Paleocene	US	Japan/late Miocene	
*Cephalotaxus*	Cephalotaxaceae	Eocene	**CN**, DE	China/Eocene	
*Hovenia*	Rhamnaceae	Eocene	CA, US	China/Miocene	

AU, Australia; CA, Canada; CN, China; DE, Germany; GE, Georgia; IE, Ireland; JP, Japan; RU, Russia; UA, Ukraine; UK, United Kingdom; US, United States; UZ, Uzbekistan; –, not found.

a Ding et al. (2018). Bold values represent two countries in EA, CN and JP are China and Japan, respectively.

### Spatial distribution of divergence times

**Figure 3:**
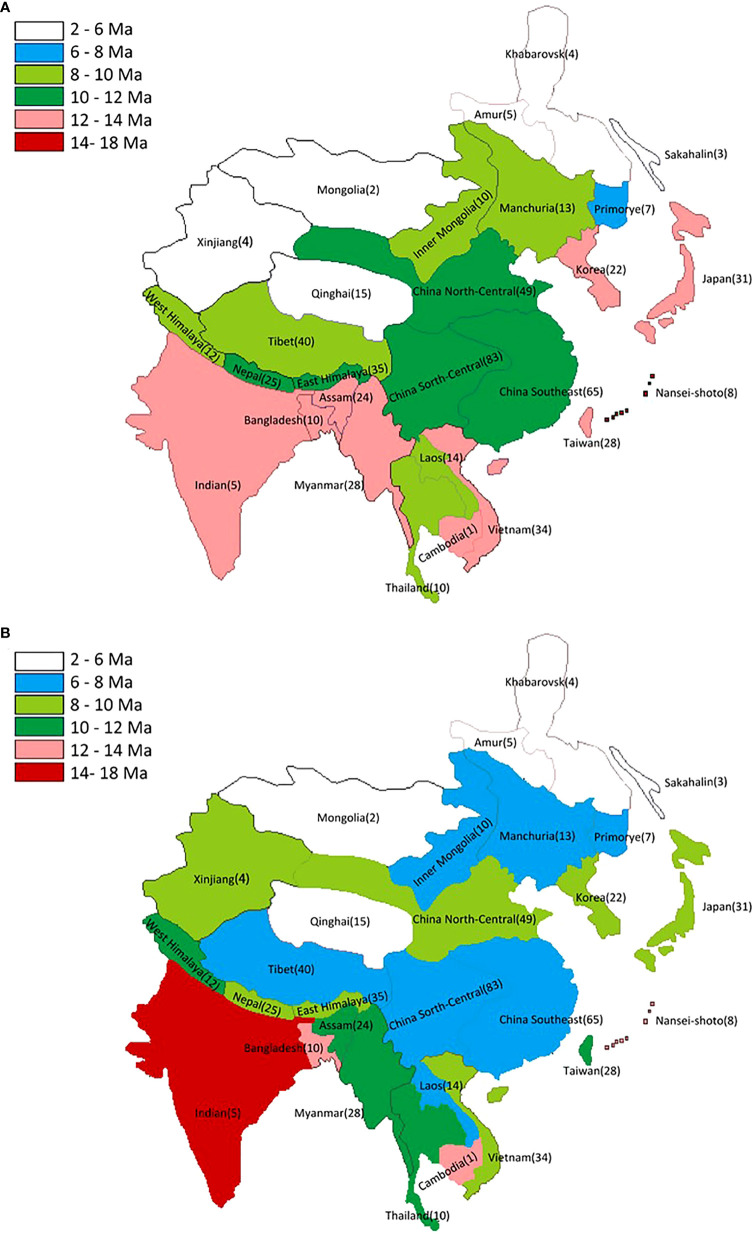
Pattern of the mean diversification times (MDTs) **(A)** and the average diversification times (ADTs) **(B)** for the East Asian endemic plant taxa. The numbers of taxa distributed in each region are labeled.

We obtain ca. 600 distribution information of the sampled 96 taxa, and the number of taxa distributed in the 29 regions ranges from one to 83 with the highest density located in south and central China (China North-Central, China South-Central, and China Southeast region) (**Figure 2** and **Supplementary Table S2**). The MDTs range from 2.4 Ma (in Khabarovsk) to 16.50 Ma (in Nansei-shoto), and a gradual decrease from coastal regions to inner mainland regions is shown. The MDTs of plants located at the ISM and the transitional region are mostly in the mid-Miocene, and those located at the EASM region are in the middle to late Miocene (**Figure 3** and **Supplementary Table S2**). Moreover, the ADTs have the youngest time in Khabarovsk (ADT = 3.22 Ma) and the oldest in India (ADT = 15.18 Ma), and the MDTs of plants located in the monsoon region are mostly in the late Miocene (**Figure 3** and **Supplementary Table S2**).

## Discussion

### Diversification of plant endemism is related to monsoon intensifications

The diversification events of over 100 (approximately 17%) endemic seed plant genera were compiled to investigate the evolution of EA plant endemism. A similar ratio of different life forms within the sampled genera compared to that of all endemic genera (Manchester et al., 2009) ensured representativeness. Diversifications (crown ages) were much more recent than origins (stem ages) in both the oldest lineage (35.91 versus 68.25 Ma) and the median values (4.52 versus 13.60 Ma) (Chen et al., 2018). The majority of the diversifications, which occurred after the late Miocene (ca. 10 Ma, **Figure 2**), also lagged behind the formation of the main body of flora, which occurred after the Miocene (ca. 23 Ma) (Chen et al., 2018; Lu et al., 2018). This time lag is similar to that found by Ramírez-Barahona et al. (2020), who shows that the origins of families were 37–56 Ma older than their diversifications across the entire angiosperm tree.

The diversification events began in the late Eocene and its accumulation did not obviously increase until after the late Miocene (**Figure 2** and **Supplementary Figure S2**), which may correlate with the uplift of mountains in southwest China (Harrison et al., 1992; Xing and Ree, 2017) or, more likely, the intensification of the Asian Monsoon (Yu et al., 2017; Wang et al., 2020). The inference receives further support from the spatial pattern. First, the distributions of sampled genera are centered in south and central China, similar to species or endemism diversity found in previous studies (Huang et al., 2016; Xu et al., 2016; Tang et al., 2018), indicating a good representativeness. Then, the MDTs and ADTs in the EASM, ISM, and transitional region fall in the mid-late Miocene (**Figures 1**, **3**). The fossils of Tertiary relict genera are mostly and widely distributed outside EA and only half of them are firstly found in EA during the Miocene, so relictualism experiences extinctions not only outside but also inside the EA, and the present species/populations are only prosperous after late Miocene when monsoon brings humid climate (Ye and Li, 2022). Moreover, it is shown that the sampled monotypic endemic taxa are mainly diversified after Pliocene, and the number of diversified genera in this period also exceeds that in the late Miocene in polytypic taxa (**Supplementary Table S1** and **Figure S2**). We suggest that the reinforcement of the EASM/ISM during the late Pliocene (An et al., 2001; Wan et al., 2006; Clift et al., 2015) and the glacial–interglacial periodic alternations in Pleistocene (Ye et al., 2017a) may also exert a greater influence on species and population divergence.

### Monsoon intensifications triggered floral evolution at various levels

Previous studies have demonstrated the influence of monsoon development on the floral evolution in EA at various taxonomic levels. Floral-level investigations of the Sino-Japanese floristic region (Chen et al., 2018), Chinese angiosperms (Lu et al., 2018), and dominant taxa in EBLFs (Xiang et al., 2016; Deng et al., 2017; Yu et al., 2017) have emphasized the climatic changes during the late Oligocene and early Miocene. Li et al. (2021) provided additional evidence by integrating modeling (SDGVM and JeDi-DGVM) and fossil data and found that the orographic evolution in northern Tibet affected vegetation and, especially, plant diversity in EA during the Neogene by altering the monsoon (both summer and winter) system. Support was gained by other studies, such as Zhang et al. (2021) who found that TP growth increased annual precipitation in East China by comparing the effects of TP growth, Paratethys Sea retreat, and global cooling on the East Asian climate change that occurred by the early Miocene. Chen and Su (2020) also found that patterns of woody dicotyledon richness in humid regions of China were mainly affected by the Asian winter monsoon by exploring the relationship between these patterns and climatic variations, especially in the Asian monsoonal climate.

Studies at the regional scale have provided further support for this idea. For example, in the Hengduan Mountains, which has the richest temperate alpine flora worldwide, Ding et al. (2020) recently found that during the early to middle Miocene and late Miocene, accelerated diversification and colonization of adjacent regions were likely driven not only by mountain development but also by Asian summer monsoon intensification. In EBLFs, Ye and Li (2022) compiled 10 radiation dates for relict evergreen genera, four diverged around the late Miocene–Pliocene boundary and four diverged during the late Pliocene, this is resulted from an increasingly humid climate after the late Miocene (An et al., 2001; Wan et al., 2006; Clift et al., 2015) and the absence of large ice sheets in the Pleistocene (Qiu et al., 2017), which allowed these genera to prosper in EBLFs. This is further supported by the origins of the dominant species in EBLFs (Ye and Li, 2022), they collected the origin time of 92 out of 291 (31.6%) dominant species and found that 70 (76.1%) species originated after ca. 8 Ma and accelerated accumulations began after 4 Ma. In Taiwan, an orogenic continental island that has high levels of endemic biological richness (Kier et al., 2009), we found that the late Pliocene–Pleistocene (3.5–2.0 Ma) was a key period for the origin of its floral endemism, as 28% (30/107) of the investigated endemic seed plants originated during this stage (Ye and Tian, unpublished data). Additionally, palynological analyses in East China have shown that well-developed mixed evergreen and deciduous broadleaved forests were strongly influenced by the EASM during the Holocene (Chen et al., 2020).

Evolution at the genus level showed close correlations with the monsoon intensification periods. Previous biogeographic studies have revealed that the intensified EASM during the late Miocene significantly increased the diversification rate in EBLFs (Deng et al., 2017; Yu et al., 2017; Wang et al., 2020). Kong et al. (2017) used environmental correlation analysis to show that the speciation rate of *Primulina* is tightly linked to the EASM strength, which provides suitable habitats for calciphiles by facilitating the dissolution and weathering of karsts. Furthermore, Kong et al. (2021) indicated that monsoon development, rather than mountain development, was probably the primary driver of the rapid diversification in *Oreocharis*. The Asian monsoon (EAM and/or IM) intensification had a similar influence on *Rhododendron* (Xia et al., 2021), *Pinus* (Liu et al., 2022), *Hedychium* (Ashokan et al., 2022), and *Typhonium* (Low et al., 2021). The transition to a monsoon climate system during the late Oligocene may have also resulted in divergence among species, such as *Prinsepia* (Ma et al., 2019).

Furthermore, monsoon intensification has promoted intraspecific population divergence (Ye et al., 2017a). Phylogeographic studies have suggested that periodic glacial–interglacial alternations are responsible for genetic divergences (Qiu et al., 2011). However, Zhang et al. (2013) found two sympatric Chinese beeches (*Fagus lucida* and *F. longipetiolata*) in subtropical China diverged during the late Miocene. These species likely migrated southward due to unsuitable environments in high latitudes; the significantly intensified EASM would have provided a major shelter. Another deciduous tree, *Cyclocarya paliurus*, originated during the early to middle Miocene and shows two peaks of lineage diversifications during the late Miocene (ca. 9.6 Ma) and late Pliocene (ca. 3.6 Ma), which coincide with the EASM intensifications (Kou et al., 2016). In evergreen plants, such as *Cephalotaxus oliveri* (Wang et al., 2014), *Quercus glauca* (Xu et al., 2015), *Tetrastigma hemsleyanum* (Wang et al., 2015), *Phoebe zhennan* (Xiao et al., 2020), *Engelhardia roxburghiana*, and *E. fenzelii* (Meng et al., 2022), their divergences and demographic expansions have also been correlated with the development of summer monsoons. In addition, the monsoon may act as a vector that facilitates long-distance dispersal (Li et al., 2012) or cause different environmental conditions owing to different monsoons (EAM or IM), which may cause/reinforce ecological adaptations (Liu et al., 2013).

### Uncertainties

The first concerns with sampling bias. Although a similar ratio of life forms in the sampled genera compare to that of all EA endemic genera is shown, limited sampling (<20%) would result in bias. Besides, an insufficient sample of species in polytypic genera or populations in monotypic genera may cause an underestimation of radiation times (Qiu et al., 2011). In the present study, 73/76 polytypic taxa contain almost all species, 15/20 monophyletic genera cover samples from its entire geographic range, and three have restricted distributions (e.g., *Gaoligongshania megalothyrsa*) (**Supplementary Table S1**), minimizing the possibility of underestimations. Finally, the spatial distribution of divergence time is based on level-3 regions of the TDWG, which are mainly divided by political boundaries in EA (**Figure 3**), and the optimal evaluations of monsoon influence are probably derived from divisions based on monsoon regions (**Figure 1**), while similar conclusions to this study are likely deduced.

The second would be caused by compiling methods. Following Chen et al. (2018) and Ye and Li (2022), we directly adopt the estimated times in previous studies. In the compiled 96 taxa, the divergence times of 19 taxa are calibrated using substitution rates that are varied among the different taxa (Drouin et al., 2008), and 12 taxa are calibrated only by one fossil or secondary point (**Supplementary Table S1**). Moreover, sequences from only one or two fragments are obtained to reconstruct the phylogeny (**Supplementary Table S1**). Although diversification rates or events are assembled after time estimations based on published or self-generated sequence data in Xing and Ree (2017) and Li et al. (2022), bias is inevitable in different dates–phylogenies using different calibrations and genetic markers. Dating a tree of life using the same calibrations and markers may exert a low resolution among genera/species (Lu et al., 2018).

The third is related to the combined influence of different geoclimate changes. The evolution of flora in EA could not solely be explained by the strengthening of the monsoons. The most important contributors to monsoon development, which are likely TP uplift and global cooling (Lu and Guo, 2014; Li et al., 2021), also exert great influence on floral evolution. Mountain uplift could lead to *in-situ* speciation, preserving dispersed species, preventing extinctions, or creating geographic isolations and so on (Favre et al., 2015; Muellner-Riehl, 2019); such as in *T. hemsleyanum*, the uplift of eastern TP may have split the species’ ancestral distribution into isolated southwestern and southern populations. Global cooling in Neogene and Pleistocene has driven the extinction of species/populations in the high-latitude region and results in southward retraction (Ye and Li, 2022). For example, the colonization to subtropical China of *C. paliurus* during the mid-Miocene might be partly explained by its sensitivity to global cooling after the Miocene optimum (Kou et al., 2016). Other geoclimate changes, such as increased precipitation during the dry (winter) season (Li et al., 2021), aridity in the Asian interior (Zhang et al., 2013), and the Holocene Climatic Optimum (Chen et al., 2020), also influence genetic divergence. Additionally, the monsoon not only increased species and genetic diversity but also decreased it by causing the disappearance of some gymnosperm genera (Liu et al., 2017).

## Conclusion and prospect

We found that the intensification of monsoon in EA triggers the evolution of its flora through the tempo-spatial pattern of diversification dates of over 100 endemic genera and by reviewing previous studies concerning flora, genus, and species levels. We call for more studies to use genomic data (Li et al., 2019; Zhou et al., 2022) and dense geographic sampling (Ding et al., 2020) to investigate the tempo-spatial evolution of endemic genera, and we believe that the influence of monsoon intensifications at different periods will be repeatedly revealed.

## Data availability statement

The original contributions presented in the study are included in the article/**Supplementary Material**. Further inquiries can be directed to the corresponding author.

## Author contributions

J-WY, BT, and D-ZL conceived the ideas, collected and analyzed the data, and wrote the manuscript. All authors contributed to the article and approved the submitted version.

## Funding

This study was supported by the CAS Strategic Priority Research Program (XDB31000000), China Postdoctoral Science Foundation (2019M663595), National Natural Science Foundation of China (NSFC) (32260056), and High-level Talents Research Start-up Fund Project of Southwest Forestry University (110222012).

## Acknowledgments

We thank the editor and the three reviewers for their insightful comments and suggestions.

## Conflict of interest

The authors declare that the research was conducted in the absence of any commercial or financial relationships that could be construed as a potential conflict of interest.

## Publisher’s note

All claims expressed in this article are solely those of the authors and do not necessarily represent those of their affiliated organizations, or those of the publisher, the editors and the reviewers. Any product that may be evaluated in this article, or claim that may be made by its manufacturer, is not guaranteed or endorsed by the publisher.

## Supplementary material

Supplementary Figure 1 Comparison of diversification dates between monotypic and polytypic endemic seed plant taxa of East Asia.

Supplementary Figure 2 Number and accumulated ratio (red line) of diversification dates of 76 polytypic endemic seed taxa of East Asia in one-million-year increments.
