# Supplementary figures and images for: Monsoon intensification in East Asia triggered the evolution of its flora
Source: Front Plant Sci. 2022 Nov 25;13:1046538. doi: 10.3389/fpls.2022.1046538 (PMC9733597; doi:10.3389/fpls.2022.1046538)

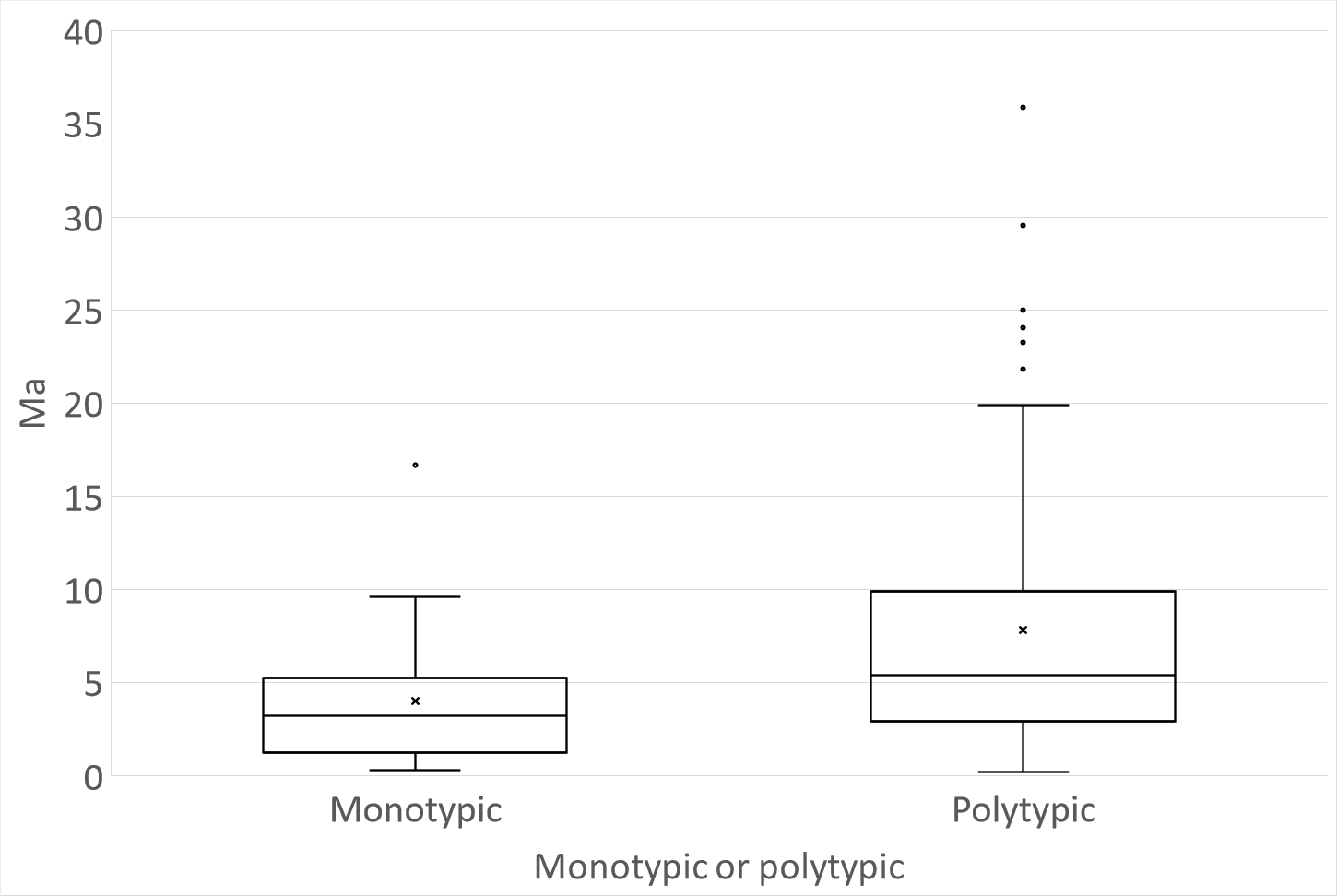

Supplement: Supplementary Figure 1 — Comparison of diversification dates between monotypic and polytypic endemic seed plant taxa of East Asia. [file Image_1.png]

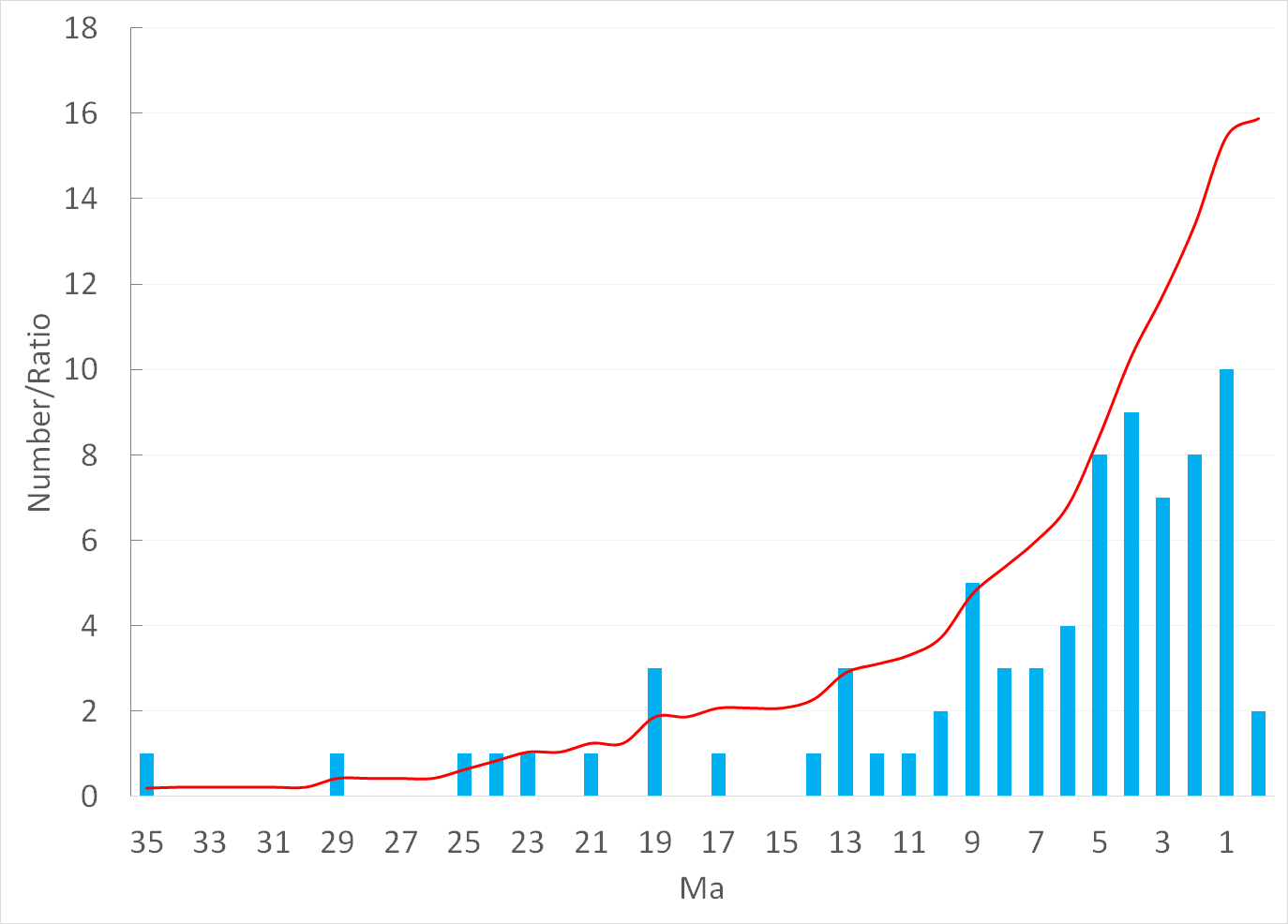

Supplement: Supplementary Figure 2 — Number and accumulated ratio (red line) of diversification dates of 76 polytypic endemic seed taxa of East Asia in one-million-year increments. [file Image_2.png]
