# Supplementary material for: Monsoon intensification in East Asia triggered the evolution of its flora
Source: Front Plant Sci. 2022 Nov 25;13:1046538. doi: 10.3389/fpls.2022.1046538 (PMC9733597; doi:10.3389/fpls.2022.1046538)
Supplement: Supplementary file 3 [file Table_1.docx]

| **Supplementary Table S1** Divergence time (median and 95% highest-posterior-density interval, HPD) and calibrations of 102 endemic seed plant genera of East Asian Flora. | | | | | | | | |
| --- | --- | --- | --- | --- | --- | --- | --- | --- |
| Family | Genus | Sample (number of sampled species/number of species of the genus, sampled species) | Marker | Calibrations | Divergence time | 95% HPD low | 95% HPD high | Reference |
| Bambusoideae | *Gaoligongshania* | 1/1, *G. megalothyrsa* | *rbcL-psaI, rpl32-trnL, rps15-ndhF, trnG-trnT, trnT-trnL, ycf4-cemA* | Four fossils and one secondary calibration | 0.04 | 0.01 | 0.15 | ([Zhang et al., 2016a](#_ENREF_68)) |
| Cupressaceae | *Cryptomeria* | 1/1, *C. japonica* | nuclear microsatellites | Substitution rate | 0.08 | 0.05 | 0.10 | ([Kimura et al., 2014](#_ENREF_18)) |
| Hydrangeaceae | *Cardiandra* | 2/2, *C. alternifolia, C. amamiohsimensis* | *rbcL, matK, trnK intron, trnS–trnG* | Substitution rate | 0.25 | 0.25 | 0.25 | ([Setoguchi et al., 2006](#_ENREF_43)) |
| Rubiaceae | *Guihaiothamnus* | 1/1, *G. acaulis* | *ndhF, rbcL, rps16, trnT-F* | Five fossils | 0.29 | 0.00 | 0.76 | ([Xie et al., 2014](#_ENREF_59)) |
| Taxodiaceae | *Glyptostrobus* | 1/1, *G. pensilis* | nuclear microsatellites | Substitution rate | 0.33 | 0.16 | 0.49 | ([Wu et al., 2019](#_ENREF_55)) |
| Ginkgoaceae | *Ginkgo* | 1/1, *G. biloba* | chloroplast genome | Eleven fossils | 0.39 | 0.22 | 0.79 | ([Hohmann et al., 2018](#_ENREF_13)) |
| Boraginaceae | *Chionocharis* | 1/1, *C. hookeri* | *atpI–atpH, rps16*, ITS | Substitution rate | 0.66 | 0.33 | 1.27 | ([Luo et al., 2016](#_ENREF_31)) |
| Leguminosae | *Piptanthus* | 3/3, *P. concolor, P. nepalensis, P. tomentosus* | ITS | Seven fossils | 0.83 | 0.83 | 0.83 | ([Zhang et al., 2015b](#_ENREF_67)) |
| Hydrangeaceae | *Platycrater* | 1/1, *P. arguta* | ITS, Tpi, nuclear microsatellites, *trnD-trnE, trnH-psbA* | Substitution rate | 0.89 | 0.51 | 1.20 | ([Qi et al., 2014](#_ENREF_38)) |
| Rutaceae | *Phellodendron* | 2/5, *P. amurense, P. chinense* | *rps16, trnL-trnF*, ETS, ITS | Two fossils and one secondary calibration | 1.11 | 0.15 | 2.81 | ([Appelhans et al., 2018](#_ENREF_1)) |
| Styracaceae | *Pterostyrax* | 2/2, *P. corymbosus, P. psilophyllus* | *atpB, matK, ndhF, rbcL, matR* | 138 fossils | 1.15 | 1.15 | 1.15 | ([Lu et al., 2018](#_ENREF_30)) |
| Brassicaceae | *Megadenia* | 3/3, *M. bardunovii, M. speluncarum, M. pigmaea* | *trnK, trnH–psbA, petG–trnP, trnS–trnG, rps16–trnQ,, rpL32–trnL, trnT–trnF* | Two secondary calibration | 1.15 | 0.65 | 1.89 | ([Artyukova et al., 2014](#_ENREF_3)) |
| Lamiaceae | *Ombrocharis+Perillula* | 1/1, *O. dulcis* and 1/1, *P. reptans* | ETS, ITS, *rbcL, matK, trnL-F, ycf1, ycf1-rps15* | Two fossils | 1.2 | 0.30 | 2.20 | ([Li et al., 2017](#_ENREF_24)) |
| Tapisciaceae | *Tapiscia* | 2/2, *T. sinensis, T. yunnanensis* | *psbA-trnH, rpl32-trnL* | Substitution rate | 1.33 | 1.02 | 1.74 | ([Zhang et al., 2015a](#_ENREF_65)) |
| Asteraceae | *Soroseris+Stebbinsia* | 3/6, *Soroseris. glomerata, S. hookeriana, S. teres* and 1/1, *Stebbinsia umbrella* | ITS | Three fossils | 1.56 | 0.53 | 2.82 | ([Zhang et al., 2011](#_ENREF_66)) |
| Gesneriaceae | *Paraisometrum* | 1/1, *P. mileense* | ITS | Substitution rate | 1.6 | 1.34 | 1.86 | ([Chen et al., 2014](#_ENREF_7)) |
| Araliaceae | *Metapanax* | 2/2, *M. delavayi, M. davidii* | *ndhF, trnL-F, rps16, atpB-rbcL, rpl16, psbA-trnH,* ITS | Two fossils | 1.62 | 1.62 | 1.62 | ([Li and Wen, 2013](#_ENREF_25)) |
| Primulaceae | *Pomatosace* | 1/1, *P. filicula* | *matK, trnH-psbA* | Substitution rate | 1.64 | 0.73 | 2.66 | ([Wang et al., 2014](#_ENREF_50)) |
| Orobanchaceae | *Phtheirospermum-1* | 1/7, *P. japonicum* | ITS, *matK, rbcL, rps2, rps16, trnK-matK, trnH-psbA,, trnL-F* | Four fossils | 1.72 | 0.29 | 3.88 | ([Yu et al., 2018](#_ENREF_61)) |
| Apiaceae | *Cyclorhiza* | 2/2, *C. waltonii, C. peucedanifolia* | ITS | Three microfossils | 1.82 | 1.82 | 1.82 | ([Banasiak et al., 2013](#_ENREF_5)) |
| Asteraceae | *Opisthopappus* | 2/2, *O. longilobus, O. taihangensis* | *trnL–trnF, ndhJ-trnL,* ITS | Substitution rate | 1.9 | 0.50 | 1.72 | ([Wang and Yan, 2014](#_ENREF_54)) |
| Pinaceae | *Keteleeria* | 8/11, *K. xerophila, K. davidiana, K. evelyniana, K. fortunei, K. pubescens, K. hainanensis, K. calcarea, K. sp* | 27 nuclear genes | Substitution rate | 1.9 | 0.40 | 3.40 | ([Chou, 2020](#_ENREF_9)) |
| Saxifragaceae | *Rodgersia* | 5/5, *R. sambucifolis, R. pinnata, R. aesculifolia var. henricii, R. aesculifolia var. aesculifolia, R. podophylla, R. nepalensis* | ITS | Secondary calibrations | 2.13 | 1.16 | 3.50 | ([Ma et al., 2017b](#_ENREF_34)) |
| Hydrangeaceae | *Kirengeshoma* | 2/2, *K. palmata, K. koreana* | *trnL–trnF, psbA–trnH, trnS–trnG* | Substitution rate | 2.25 | 1.70 | 2.80 | ([Qiu et al., 2009](#_ENREF_39)) |
| Brassicaceae | *Solms-laubachia* | 10/10, *S. minor, S. platycarpa, S. prolifera, S. pulcherrima, S. pumila, S. retropilosa, S. stewartii, S. sunhangiana, S. xerophyta, S. zhongdianensis* | Chs | One pollen calibration | 2.31 | 1.42 | 3.68 | ([Yue et al., 2009](#_ENREF_63)) |
| Asteraceae | *Tugarinovia* | 1/1, *T. mongolica* | *psbA-trnH, psbK-psbI* | Substitution rate | 2.5 | 1.21 | 4.23 | ([Zhao et al., 2019](#_ENREF_72)) |
| Sapindaceae | *Koelreuteria* | 2/2, *K. paniculata, K. elegans* | *atpB, matK, ndhF, rbcL, matR* | 138 fossils | 2.57 | 2.57 | 2.57 | ([Lu et al., 2018](#_ENREF_30)) |
| Asteraceae | *Syncalathium-1* | 6/7~8, *S. pilosum, S. roseum, S. kawaguchi, S. chrysocephalum, S. disciforme, S. qinghaiense* | ITS | Three fossils | 2.64 | 1.78 | 4.33 | Zhang et al., 2011, TAXON |
| Brassicaceae | *Baimashania* | 2/2, *B. pulvinata, B. wangii* | *trnL–trnF* | One secondary calbrations | 2.72 | 0.60 | 5.53 | ([Zhang et al., 2011](#_ENREF_66)) |
| Asteraceae | *Nannoglottis* | 7/~8, *N. delavayi, N. gynura, N. hieraciphylla, N. latisquama, N. macrocarpa, N. ravida, N. yuennanensis* | *trnL-F*, ITS | Substitution rate | 2.89 | 2.41 | 3.37 | ([Liu et al., 2002](#_ENREF_27)) |
| Dipentodontaceae | *Dipentodon* | 2/2, *D. longipedicellatus, D. sinicus* | *psbA-trnH, trnQ-rps16* | Substitution rate | 2.97 | 2.97 | 2.97 | ([Yuan et al., 2008](#_ENREF_62)) |
| Saxifragaceae | *Oresitrophe+Mukdenia* | 1/1, *O. rupifraga* and 1/1, *M. rosii* | *rbcL, trnL-trnF, matK* | One fossil | 3.1 | 1.27 | 5.32 | ([Deng et al., 2015](#_ENREF_11)) |
| Asteraceae | *Syncalathium-2* | 1/7~8, *S. souliei* | ITS | Three fossils | 3.23 | 1.52 | 5.30 | ([Zhang et al., 2011](#_ENREF_66)) |
| Cupressaceae | *Taiwania* | 1/1, *T. cryptomerioides* | LFY, NLY, *matK* | Two fossils and substitution rate | 3.31 | 2.51 | 4.12 | ([Chou et al., 2011](#_ENREF_10)) |
| Araliaceae | *Fatsia* | 2/2, *F. japonica, F. polycarpa* | *atpB-rbcL, psbA-trnH, trnL-trnF, nadhF, rpL16, rpS16* | Two fossils | 3.53 | 3.53 | 3.53 | ([Valcárcel et al., 2014](#_ENREF_49)) |
| Orchidaceae | *Cremastra* | 2/3, *C. appendiculata, C. unguiculata* | *atpB, matK, ndhF, rbcL, matR* | 138 fossils | 3.64 | 3.64 | 3.64 | ([Lu et al., 2018](#_ENREF_30)) |
| Sapotaceae | *Eberhardtia* | 2/2, *E. tonkinensis, E. aurata* | ITS | Three fossils | 3.64 | 3.64 | 3.64 | ([Armstrong et al., 2014](#_ENREF_2)) |
| Lardizabalaceae | *Akebia+Archakebia* | 1/1, *Archakebia apetala* and 1/4, *Akebia quinata* | *atpB, matK, ndhF, rbcL* | 138 fossils | 3.67 | 3.67 | 3.67 | ([Lu et al., 2018](#_ENREF_30)) |
| Taxaceae | *Pseudotaxus* | 1/1, *P. chienii* | low-copy nuclear genes | Substitution rate | 3.68 | 2.60 | 4.63 | ([Kou et al., 2019](#_ENREF_21)) |
| Papaveraceae | *Hylomecon* | 2/2, *H. japonica, H. vernalis* | *atpB, matK, ndhF, rbcL, matR* | 138 fossils | 3.87 | 3.87 | 3.87 | ([Lu et al., 2018](#_ENREF_30)) |
| Arecaceae | *Guihaia* | 2/2, *G. argyrata, G. grossifibrosa* | CISP4, CISP5 *RPB2, matK, ndhF, trnDT* | Three fossils | 3.92 | 1.32 | 7.01 | ([Bacon et al., 2012](#_ENREF_4)) |
| Rosaceae | *Docynia* | 2/2, *D. indica, D. delavagi* | *trnG–trnS, rpl20–rps12, trnC–ycf6, psbA–trnH,, trnH–rpl2, trnL–trnF, trnK-matK, rpl16 intron, rps16, atpB–rbcL, rbcL,* ITS | Two fossils and one secondary calibrations | 4 | 2.00 | 6.00 | ([Eugenia and Donoghue, 2012](#_ENREF_12)) |
| Berberidaceae | *Dysosma* | 7/~7, *D. tsayuensis, D. aurantiocaulis, D. delavayi, D. versipellis, D. pleiantha, D. difformis, D. majoensis* | *trnL–trnF, trnL–ndhJ, trnS–trnfM* | Substitution rate | 4.14 | 2.74 | 5.44 | ([Wang et al., 2017](#_ENREF_53)) |
| Campanulaceae | *Homocodon* | 2/2, *H. pedicellatus, H. brevipes* | *atpB, matK, rbcL, trnL–trnF* | One fossil | 4.21 | 1.52 | 7.02 | ([Zhou et al., 2013](#_ENREF_74)) |
| Papaveraceae | *Macleaya* | 2/2, *M. microcarpa, M. cordata* | *atpB, matK, ndhF, rbcL, matR* | 138 fossils | 4.24 | 4.24 | 4.24 | ([Lu et al., 2018](#_ENREF_30)) |
| Asteraceae | *Notoseris* | 6/6, *N. macilenta, N. henryi, N. khasiana, N. triflora, N. yakoensis, N. scandens* | ITS | One fossil and one secondary calibration | 4.3 | 2.10 | 6.60 | ([Kilian et al., 2017](#_ENREF_17)) |
| Solanaceae | *Anisodus* | 4/4, *A. acutangulus, A. carniolicoides, A. luridus, A. tanguticus* | *ndhF, atpB, rbcL, trnH, rps16-trnK, trnC-psbM* | Two fossils | 4.35 | 1.63 | 7.71 | ([Tu et al., 2010](#_ENREF_48)) |
| Linaceae | *Anisadenia* | 2/2, *A. pubdescens, A. saxatilis* | *atpB, matK, ndhF, rbcL, matR* | 138 fossils | 4.5 | 4.50 | 4.50 | ([Lu et al., 2018](#_ENREF_30)) |
| Apiaceae | *Changium+Chuanminshen* | 1/1, *Changium Smyrnioides* and 1/1, *Chuanminshen violaceum* | ITS | Three microfossils | 4.53 | 4.53 | 4.53 | ([Banasiak et al., 2013](#_ENREF_5)) |
| Leguminosae | *Salweenia* | 2/2, *S. wardii, S. bouffordiana* | *trnL-F, rpl32-trnL, psbA-trnH, trnS-G* | Substitution rate | 4.54 | 3.03 | 6.06 | ([Yue et al., 2011](#_ENREF_64)) |
| Davidiaceae | *Davidia* | 1/1, *D. involucrata* | *trnL–rpl32F, trnS–trnG* | Two fossils | 4.81 | 3.43 | 6.34 | ([Ma et al., 2015](#_ENREF_33)) |
| Rubiaceae | *Emmenopterys* | 1/1, *E. henryi* | *psbA–trnH, trnL–trnF, trnT–trnL* | One secondary calibration | 5.06 | 1.69 | 8.91 | ([Zhang et al., 2016b](#_ENREF_69)) |
| Cercidiphyllaceae | *Cercidiphyllum* | 2/2, *C. japonicum, C. magnificum* | *atpB, matK, rbcL* | Two fossils | 5.32 | 1.93 | 9.25 | ([Qi et al., 2012](#_ENREF_37)) |
| Taxodiaceae | *Cunninghamia* | 2/2, *C. lanceolata, C. konishii* | *rbcL, matK,* 18S, PHYP | Sixteen fossils | 5.41 | 1.22 | 8.25 | ([Leslie et al., 2012](#_ENREF_22)) |
| Asparagaceae | *Heteropolygonatum* | 4/7, *H. altelobatum, H. ginfushanicum, H. pendulum, H. roseolum* | ITS, *psbA-trnH, trnC-petN, rbcL, matK* | Two secondary calibrations | 5.42 | 1.54 | 8.57 | ([Wang et al., 2016](#_ENREF_52)) |
| Eupteleaceae | *Euptelea* | 2/2, *E. pleiosperma, E. polyandra* | *rbcL, matK,* 26S nrDNA | Four fossils | 5.46 | 1.23 | 10.87 | ([Cao et al., 2016](#_ENREF_6)) |
| Calycanthaceae | *Chimonanthus* | 7/7, *C. campanulatus, C. praecox, C. grammatus, C. salicifolius, C. nitens, C. zhejiangensis, C. gramatus* | ITS, *trnL–F, trnC–D* | One fossil and one secondary calibration | 5.5 | 4.35 | 6.65 | ([Zhou et al., 2006](#_ENREF_73)) |
| Trochodendraceae | *Trochodendron* | 1/1, *T. aralioides* | *petG-trnP, petA-psbJ* | Substitution rate | 5.5 | 2.70 | 8.30 | ([Huang and Lin, 2006](#_ENREF_15)) |
| Solanaceae | *Physochlaina+Przewalski* | 1/6, *Physochlaina praealta* and 1/1, *Przewalski tangutica* | *ndhF, atpB, rbcL, trnH, rps16-trnK, trnC-psbM* | Two fossils | 5.75 | 2.35 | 9.44 | ([Tu et al., 2010](#_ENREF_48)) |
| Lamiaceae | *Perilla+Keiskea* | 1/1, *P. frutescens* and 5/7, *K. australis, K. elsholtzioides, K. glandulosa, K. japonica, K. macrobracteata* | ETS, ITS, *rbcL, matK, trnL-F, ycf1, ycf1-rps15* | Two fossils | 5.81 | 3.98 | 7.77 | ([Li et al., 2017](#_ENREF_24)) |
| Brassicaceae | *Orychophragmus* | 6/6, *O. violaceus, O. hupehensis, O. longisiliqus, O. taibaiensis, O. zhongtiaoshanus, O. diffusus* | chloroplast genome | Substitution rate | 5.91 | 4.22 | 7.61 | ([Hu et al., 2016](#_ENREF_14)) |
| Orobanchaceae | *Phtheirospermum-2* | 6/7, *P. tenuisectum, P. muliense, P. nigrescens, P. cylindrica, P. duclouxii, P. sp1, P. sp2* | ITS, *matK, rbcL, rps2, rps16, trnK-matK, trnH-psbA, trnL-F* | Four fossils | 6.16 | 2.91 | 10.21 | ([Yu et al., 2018](#_ENREF_61)) |
| Orchidaceae | *Holcoglossum* | 15/15, *H. amesianum,H. flavescens, H. himalaicum, H. kimballianum, H. lingulatum, H. nagalandensis, H. nujiangense, H. omeiense, H. pumilum, H. quasipinifolium, H. rupestre, H. sinicum, H. subulifolium, H. wangii, H. weixiense* | ITS, *atpH-I, matK, psbA-trnH, psbK-I, rbcL, rpoB, rpoC1, rpS12-rpL20, trnL-F, trnS-fM, trnS-G* | One secondary calibration | 6.33 | 3.30 | 10.60 | ([Zhao et al., 2020](#_ENREF_70)) |
| Caprifoliaceae | *Triplostegia* | 2/2, *T. glandulifera,  T. grandiflora* | *psbK–psbI, rpl20–rps12, trnH–psbA, trnL–F, trnS–trnG* | Three fossils and one secondary calibration | 6.49 | 2.50 | 13.60 | ([Niu et al., 2018](#_ENREF_36)) |
| Arecaceae | *Trachycarpus* | 4/9, *T. fortunei, T. takil, T. nanus, T. martianus* | CISP4, CISP5 RPB2, *matK, ndhF, trnDT* | One secondary calbrations and 5 fossils | 6.63 | 3.72 | 12.11 | ([Bacon et al., 2012](#_ENREF_4)) |
| Lardizabalaceae | *Sargentodoxa* | 1/1, *S. cuneata* | *matK* | Four fossils | 7.29 | 2.86 | 13.26 | ([Tian et al., 2015](#_ENREF_47)) |
| Liliaceae | *Cardiocrinum* | 3/3, *C. cathayanum, C. cordatum, C. giganteum* | *atpI-atpH, trnT-trnL, atpB-rbcL, rpl32- trnL, ndhA*, AP, AT103, XDH | One secondary calibration | 7.32 | 5.70 | 8.95 | ([Yang et al., 2017](#_ENREF_60)) |
| Anacardiaceae | *Dobinea* | 2/2, *D. vulgaris, D. delavayi* | *atpB, matK, ndhF, rbcL, matR* | 138 fossils | 7.65 | 7.65 | 7.65 | ([Lu et al., 2018](#_ENREF_30)) |
| Apiaceae | *Notopterygium* | 4/6, *N. incisum, N. franchetii, N. oviforme, N. forrestii* | *trnS-trnG, matK, rbcL, ndhF* | Substitution rate | 7.82 | 3.12 | 15.93 | ([Liu et al., 2019](#_ENREF_28)) |
| Celastraceae | *Tripterygium* | 3/3, *T. wilfordii, T. regelii, T. hypoglaucum* | *psbA-trnH, rpl32-trnL, trnL-trnF* | Two fossils | 8.13 | 5.38 | 12.81 | ([Ma et al., 2017a](#_ENREF_32)) |
| Lamiaceae | *Siphocranion* | 2/2, *S. nudipes, S. macranthus* | *atpB, matK, ndhF, rbcL, matR* | 138 fossils | 8.74 | 8.71 | 8.71 | ([Lu et al., 2018](#_ENREF_30)) |
| Ranunculaceae | *Urophysa* | 2/2, *U. rockii, U. henryi* | *psbA-trnH, trnL-trnF* | Two secondary calibrations | 8.86 | 6.24 | 11.00 | ([Xie et al., 2017](#_ENREF_58)) |
| Campanulaceae | *Cyananthus* | 17/~20, *C. pedunculatus, C. cordifolius, C. lobatus, C. microphyllus, C. sherriffii, C. sericeus, C. longiflorus, C. macrocalys, C. flavus ssp. Montanus, C. flavus ssp. flavus, C. formosus, C. delavayi, C. incanus, C. dolichosceles, C. inflatus, C. lichiangensis, C. hookeri* | *atpB, matK, rbcL, trnL–trnF* | One fossil | 9.3 | 5.98 | 12.46 | ([Zhou et al., 2013](#_ENREF_74)) |
| Rubiaceae | *Damnacanthus* | 8/12, *D. giganteus, D. henryi, D. indicus, D. labordei, D. macrophyllus, D. major, D. officinarum, D. hananensis* | ITS, *atpB-rbcL, rbcL, rps16, trnL-F* | Four fossils and one secondary calibration | 9.44 | 5.42 | 14.40 | ([Huang et al., 2014](#_ENREF_16)) |
| Trochodendraceae | *Tetracentron* | 1/1, *T. sinense* | *petG–trnP, psbK–psbI, rps2, rpl16 intron, psbA–trnH, rpl32–trnL, petA–psbJ, petL–psbE* | One secondary calibration | 9.6 | 2.20 | 27.00 | ([Sun et al., 2014](#_ENREF_46)) |
| Zingiberaceae | *Cautleya* | 2/2, *C. gracilis, C. spicata* | nrITS, *trnK* | One secondary calibration | 9.61 | 9.61 | 9.61 | ([Zhao et al., 2016](#_ENREF_71)) |
| Caprifoliaceae | *Dipelta* | 3/3, *D. yunnanensis, D. floribunda, D. elegans* | ITS, *matK, rbcL, trnL-F, trnS-G, ndhA, petB-D, psbA-trnH, psbM-trnD, trnL-rpl32* |  | 9.92 | 1.83 | 20.85 | ([Wang et al., 2015](#_ENREF_51)) |
| Betulaceae | *Ostryopsis* | 3/3, *Q. intermedia, O. davidiana, O. nobilis* | ITS, *matK, rbcL,* 11 nuclear genes, 20 nuclear microsatellites | Two fossils | 9.95 | 6.00 | 11.24 | ([Liu et al., 2014](#_ENREF_26)) |
| Urticaceae | *Nanocnide* | 2/2, *N. japonica, N. lobata* | *trnL-trnF, rpl14-rps8-infA-rpl36, matK, rbcL*, ITS, 18S, *matR* | Five fossils | 10.6 | 4.10 | 17.90 | ([Wu et al., 2018](#_ENREF_56)) |
| Aceraceae | *Dipteronia* | 2/2, *D. dyerana, D. sinensis* | *atpB, matK, ndhF, rbcL, matR* | 138 fossils | 10.97 | 10.97 | 10.97 | ([Lu et al., 2018](#_ENREF_30)) |
| Vitaceae | *Yua* | 2/2, *Y. thomsonii, Y. austro-orientalis* | *trnLF, the rps16 intron, atpB-rbcL, trnH-psbA, trnC-petN*, GAI1, ITS | Three fossils and one secondary calibration | 11.9 | 2.10 | 29.12 | ([Liu et al., 2016](#_ENREF_29)) |
| Taxaceae | *Amentotaxus* | 4/~6, *A. argotaenia, A. formosana, A. yunnanensis, A. poilanei* | LFY, NLY, *matK*, *rbcL* |  | 12.14 | 3.90 | 22.40 | ([Leslie et al., 2012](#_ENREF_22)) |
| Cucurbitaceae | *Hemsleya* | 23/~26, *H. graciliflora, H. zhejiangensis, H. turbinate, H. sphaerocarpa, H. pengxianensis, H. panlongqi, H. panacis-scandens, H. omeiensis, H. mitrata, H. macrosperma, H. macrocarpa, H. longicarpa, H. lijiangensis, H. gigantha, H. endecaphylla, H. ellipsoidea, H. dulongjiangensis, H. dipterygia, H. delavayi, H. chinensis, H. chengyihana, H. carnosiflora, H. amabilis* | ITS, *rpl16, trnH-psbA, trnL* | One secondary calibration | 13 | 6.00 | 21.11 | ([Schaefer et al., 2009](#_ENREF_41);[Li et al., 2010](#_ENREF_23)) |
| Altingiaceae | *Semiliquidambar* | 2/3, *S. cathayensis, S. chingii* | *atpB, matK, ndhF, rbcL, matR* | 138 fossils | 13.74 | 13.74 | 13.74 | ([Lu et al., 2018](#_ENREF_30)) |
|  |  |  |  |  |  |  |  |  |
| Gesneriaceae | *Primulina* | 159 described and 31 undescribed species | *atpB-rbcL, rpl32-trnL, trnL-trnF, rpoBtrnC, trnC-petN, ndhA_intron, ndhH-rps15-ycf1, ycf1_1, ycf1_2*, 7FR, 13FR, 97FR, 117FR, 155FR, 165FR, 166FR, 248FR, 302FR, 383FR | One secondary calibration | 14.14 | 10.79 | 19.09 | ([Kong et al., 2017](#_ENREF_19)) |
| Juglandaceae | *Cyclocarya* | 1/1, *C. paliurus* | *atpB–rbcL, psbA–trnH* | Four fossils | 16.69 | 8.42 | 27.86 | ([Kou et al., 2016](#_ENREF_20)) |
| Ericaceae | *Enkianthus* | 8/13, *E. campanulatus, E. cernuus, E. chinensis, E. deflexus, E. nudipes, E. perulatus, E. quinqueflorus, E. subsessilis* | *rbcL, matK* | Eighteen fossils | 17.33 | 17.33 | 17.33 | ([Schwery et al., 2015](#_ENREF_42)) |
| Cephalotaxaceae | *Cephalotaxus* | 11/~13, *C. lanceolata, C. fortunei, C. sinensis, C. latifolia, C. griffithii, C. hainanensis, C. mannii, C. wilsoniana, C. harringtonia, C. oliveri, C. koreana* | *rbcL, matK,* 18S, *PHYP* | Sixteen fossils | 19.48 | 12.18 | 27.74 | ([Leslie et al., 2012](#_ENREF_22)) |
| Araceae | *Pinellia* | 7/9, *P. cordata, P. ternata, P. yaoluopingensis, P. polyphylla, P. pedatisecta, P. integrifolia, P. tripatita* | *trnl, matK, rbcL* |  | 19.82 | 11.39 | 29.01 | ([Renner and Zhang, 2004](#_ENREF_40);[Lu et al., 2018](#_ENREF_30)) |
| Liliaceae | *Tricyrtis* | 15/18, *T. nana, T. ohsumiensis, T. perfoloata, T. flava, T. hirta, T. amethyystina, T. formosana, T. maculata, T. latifolia, T. affinis, T. macropoda, T. macranthopsis, T. setouchiensis, T. ishiiana, T. suzukii* | *rps16* | Substitution rate | 19.91 | 18.93 | 20.88 | ([Sophia and Jury, 2011](#_ENREF_44)) |
| Ranunculaceae | *Dichocarpum* | 15/19, *D. adiantifolium, D. arisanense, D. auriculatum, D. basilare, D. carinatum, D. dalzielii, D. fargesii, D. franchetii, D. hypoglaucum, D. lobatipetalum, D. nipponicum, D. pterigionocaudatum, D. stoloniferum, D. sutchuenense, D. trachyspermum, D. trifoliolatum, D. wuchuanense* | *matK, trnL-F, trnH-psbA*, ITS | Two second calibrations | 21.86 | 16.36 | 27.32 | ([Xiang et al., 2017](#_ENREF_57)) |
| Zingiberaceae | *Roscoea* | 15/~21, *R. australis, R. cautleyoides, R. debilis, R. forrestii, R. humeana, R. kunmingensis, R. praecox, R. schneideriana, R. tibetica, R. wardii, R. alpina, R. auriculata, R. capitata, R. purpurea, R. tumjensis* | nrITS, *trnK* | One secondary calibration | 23.28 | 13.20 | 37.80 | ([Zhao et al., 2016](#_ENREF_71)) |
| Rosaceae | *Prinsepia* | 4/4, *P. utilis, P. scandens, P. uniflora, P. sinensis* | ITS | Two fossils | 24.06 | 11.41 | 38.75 | ([Ma et al., 2019](#_ENREF_35)) |
| Rosaceae | *Chaenomeles* | 4/5, *C. speciosa, C. cathayensis, C. lagenari, C. japonica* | *trnG–trnS, rpl20–rps12, trnC–ycf6, psbA–trnH, trnH–rpl2, trnL–trnF, trnK + matK, rpl16 intron, rps16 intron, atpB–rbcL, rbcL,* ITS | One secondary calibration and two fossils | 25 | 19.00 | 30.00 | ([Eugenia and Donoghue, 2012](#_ENREF_12)) |
| Caprifoliaceae | *Abelia* | 6/6, *A. chinensis, A. forrestii,, A. × grandiflora, A. macrotera, A. schumannii, A. uniflora*) | nuclear gene | One secondary calibration and one fossil | 29.56 | 21.64 | 33.76 | ([Sun et al., 2021](#_ENREF_45)) |
| Rhamnaceae | *Hovenia* | 3/3, *H. trichocarpa, H. dulcis, H. acerba*) | ITS, *trnL-F, rbcL* | One fossil | 35.91 | 24.23 | 49.31 | ([Chen et al., 2017](#_ENREF_8)) |

Reference

Appelhans, M.S., Reichelt, N., Groppo, M., Paetzold, C., and Wen, J. (2018). Phylogeny and biogeography of the pantropical genus *Zanthoxylum* and its closest relatives in the proto-Rutaceae group (Rutaceae). *Mol Phylogenet Evol* 126**,** 31-44. doi: [10.1016/j.ympev.2018.04.013](https://doi.org/10.1016/j.ympev.2018.04.013)

Armstrong, K.E., Stone, G.N., Nicholls, J.A., Valderrama, E., Anderberg, A.A., Smedmark, J., Gautier, L., Naciri, Y., Milne, R., and Richardson, J.E. (2014). Patterns of diversification amongst tropical regions compared: a case study in Sapotaceae. *Front Genet* 5**,** 362. doi: [10.3389/fgene.2015.00086](https://doi.org/10.3389/fgene.2015.00086)

Artyukova, E.V., Kozyrenko, M.M., Boltenkov, E.V., and Gorovoy, P.G. (2014). One or three species in *Megadenia* (Brassicaceae): Insight from molecular studies. *Genetica* 142**,** 337-350. doi: 10.1007/s10709-014-9778-1

Bacon, C.D., Baker, W.J., and Simmons, M.P. (2012). Miocene dispersal drives island radiations in the palm tribe Trachycarpeae (Arecaceae). *Syst Biol* 61**,** 426-442.

Banasiak, Ł., Piwczyński, M., Uliński, T., Downie, S.R., Watson, M.F., Shakya, B., and Spalik, K. (2013). Dispersal patterns in space and time: a case study of Apiaceae subfamily Apioideae. *J Biogeogra* 40**,** 1324-1335. doi: [10.1111/jbi.12071](https://doi.org/10.1111/jbi.12071)

Cao, Y.N., Comes, H.P., Sakaguchi, S., Chen, L.Y., and Qiu, Y.X. (2016). Evolution of East Asia’s Arcto-Tertiary relict *Euptelea* (Eupteleaceae) shaped by Late Neogene vicariance and Quaternary climate change. *BMC Evol Biol* 16**,** 1-17. doi: 10.1186/s12862-016-0636-x

Chen, W.H., Shui, Y.M., Yang, J.B., Wang, H., Nishii, K., Wen, F., Zhang, Z.R., and Möller, M. (2014). Taxonomic status, phylogenetic affinities and genetic diversity of a presumed extinct genus, *Paraisometrum* W.T. Wang (gesneriaceae) from the karst regions of Southwest China. *PloS ONE* 9**,** e107967. doi: [10.1371/journal.pone.0107967](https://doi.org/10.1371/journal.pone.0107967)

Chen, Y.S., Meseguer, A.S., Godefroid, M., Zhou, Z., Zhang, J.W., Deng, T., Kim, J.H., Nie, Z.L., Liu, Y.S., and Sun, H. (2017). Out-of-India dispersal of *Paliurus* (Rhamnaceae) indicated by combined molecular phylogenetic and fossil evidence. *Taxon* 66**,** 1-13.

Chou, S.Y. (2020). Genetic divergence between *Keteleeria* species (Pinaceae) using multilocus analysis. *Unpublished*.

Chou, Y.W., Thomas, P.I., Ge, X.J., Lepage, B.A., and Wang, C.N. (2011). Refugia and phylogeography of Taiwania in East Asia. *J Biogeogr* 38**,** 1992-2005. doi: [10.1111/j.1365-2699.2011.02537.x](https://doi.org/10.1111/j.1365-2699.2011.02537.x)

Deng, J.B., Drew, B.T., Mavrodiev, E.V., Gitzendanner, M.A., Soltis, P.S., and Soltis, D.E. (2015). Phylogeny, divergence times, and historical biogeography of the angiosperm family Saxifragaceae. *Mol Phylogenet Evol* 83**,** 86-98. doi: [10.3732/ajb.1100294](https://doi.org/10.3732/ajb.1100294)

Eugenia, Y.Y.L., and Donoghue, M.J. (2012). Expanded phylogenetic and dating analyses of the apples and their relatives (Pyreae, Rosaceae). *Mol Phylogenet Evol* 63**,** 230-243. doi: [10.1016/j.ympev.2011.10.005](https://doi.org/10.1016/j.ympev.2011.10.005)

Hohmann, N., Wolf, E.M., Rigault, P., Zhou, W.B., Kiefer, M., Zhao, Y.P., Fu, C.X., and Koch, M.A. (2018). *Ginkgo biloba*’s footprint of dynamic Pleistocene history dates back only 390,000 years ago. *BMC Genomics* 19**,** 299. doi: [10.1186/s12864-018-4673-2](https://doi.org/10.1186/s12864-018-4673-2)

Hu, H., Hu, Q.J., Al-Shehbaz, I.A., Luo, X., Zeng, T.T., Guo, X.Y., and Liu, J.Q. (2016). Species delimitation and interspecific relationships of the genus *Orychophragmus* (brassicaceae) inferred from whole chloroplast genomes. *Front Plant Sci* 7**,** 1826. doi: [10.3389/fpls.2016.01826](https://doi.org/10.3389/fpls.2016.01826)

Huang, S.F., and Lin, T.P. (2006). Migration of *Trochodendron aralioides* (Trochodendraceae) in Taiwan and its adjacent areas. *J Biogeogr* 31**,** 1251&ndash;1259.

Huang, W.P., Sun, H., Deng, T., Razafimandimbison, S.G., Nie, Z.L., and Wen, J. (2014). Molecular phylogenetics and biogeography of the eastern Asian–eastern North American disjunct *Mitchella* and its close relative *Damnacanthus* (Rubiaceae, Mitchelleae). *Bot J Linn Soc* 171**,** 395-412. doi: [10.1111/j.1095-8339.2012.01321.x](https://doi.org/10.1111/j.1095-8339.2012.01321.x)

Kilian, N., Sennikov, A., Wang, Z.H., Gemeinholzer, B., and Zhang, J.W. (2017). Sub-paratethyan origin and middle to late miocene principal diversification of the Lactucinae (compositae: Cichorieae) inferred from molecular phylogenetics, divergence-dating and biogeographic analysis. *TAXON* 66**,** 675-703. doi: [10.12705/663.9](https://doi.org/10.12705/663.9)

Kimura, M.K., Uchiyama, K., Nakao, K., Moriguchi, Y., San Jose-Maldia, L., and Tsumura, Y. (2014). Evidence for cryptic northern refugia in the last glacial period in *Cryptomeria japonica*. *Ann Bot-London* 114**,** 1687-1700. doi: [10.1093/aob/mcu197](https://doi.org/10.1093/aob/mcu197)

Kong, H.H., Condamine, F.L., Harris, A., Chen, J.L., Pan, B., Möller, M., Hoang, V.S., and Kang, M. (2017). Both temperature fluctuations and East Asian monsoons have driven plant diversification in the karst ecosystems from southern China. *Mol Ecol* 26**,** 6414-6429. doi: [10.1111/mec.14367](https://doi.org/10.1111/mec.14367)

Kou, Y.X., Cheng, S.M., Tian, S., Li, B., Fan, D.M., Chen, Y.J., Soltis, D.E., Soltis, P.S., and Zhang, Z.Y. (2016). The antiquity of *Cyclocarya paliurus* (Juglandaceae) provides new insights into the evolution of relict plants in subtropical China since the late Early Miocene. *J Biogeogr* 43**,** 351-360. doi: [10.1111/jbi.12635](https://doi.org/10.1111/jbi.12635)

Kou, Y.X., Zhang, L., Fan, D.M., Cheng, S.M., Li, D.Z., Hodel, R.G.J., and Zhang, Z.Y. (2019). Evolutionary history of a relict conifer, *Pseudotaxus chienii* (Taxaceae), in southeast China during the late Neogene: old lineage, young populations. *Ann Bot-London* 125**,** 105-117. doi: [10.1093/aob/mcz153](https://doi.org/10.1093/aob/mcz153)

Leslie, A.B., Beaulieu, J.M., Rai, H.S., Crane, P.R., Donoghue, M.J., and Mathews, S. (2012). Hemisphere-scale differences in conifer evolutionary dynamics. *P Nat Acad Sci USA* 109**,** 16217-16221. doi: [10.1073/pnas.1213621109](https://doi.org/10.1073/pnas.1213621109)

Li, H.T., Yang, J.B., Li, D.Z., Möller, M., and Shah, A. (2010). A molecular phylogenetic study of *Hemsleya* (Cucurbitaceae) based on ITS, rpl16, trnH-psbA, and trnL DNA sequences. *Plant Syst Evol* 285**,** 23-32. doi: 10.1007/s00606-009-0252-y

Li, P., Qi, Z.C., Liu, L.X., Ohi-Toma, T., Lee, J., Hsieh, T.H., Fu, C.X., Cameron, K.M., and Qiu, Y.X. (2017). Molecular phylogenetics and biogeography of the mint tribe Elsholtzieae (Nepetoideae, Lamiaceae), with an emphasis on its diversification in East Asia. *Sci Rep-UK* 7**,** 2057-2057. doi: 10.1038/s41598-017-02157-6

Li, R., and Wen, J. (2013). Phylogeny and biogeography of *Dendropanax* (araliaceae), an amphi-pacific disjunct genus between tropical/subtropical asia and the neotropics. *Syst Bot* 38**,** 536-551. doi: [10.1600/036364413x666606](https://doi.org/10.1600/036364413x666606)

Liu, B.B., Abbott, R.J., Lu, Z.Q., Tian, B., and Liu, J.Q. (2014). Diploid hybrid origin of *Ostryopsis intermedia* (Betulaceae) in the Qinghai-Tibet Plateau triggered by Quaternary climate change. *Mol Ecol* 23**,** 3013-3027. doi: [10.1111/mec.12783](https://doi.org/10.1111/mec.12783)

Liu, J.Q., Gao, T.G., Chen, Z.D., and Lu, A.M. (2002). Molecular phylogeny and biogeography of the Qinghai-Tibet Plateau endemic *Nannoglottis* (Asteraceae). *Mol Phylogenet Evol* 23**,** 307-325. doi: [10.1016/s1055-7903(02)00039-8](https://doi.org/10.1016/s1055-7903(02)00039-8)

Liu, M.L., He, Y.L., López-Pujol, J., Jia, Y., and Li, Z.H. (2019). Complex population evolutionary history of four cold-tolerant *Notopterygium* herb species in the Qinghai-Tibetan Plateau and adjacent areas. *Heredity* 123**,** 242-263. doi: [10.1038/s41437-019-0186-2](https://doi.org/10.1038/s41437-019-0186-2)

Liu, X.Q., Ickert-Bond, S.M., Nie, Z.L., Zhou, Z., Chen, L.Q., and Wen, J. (2016). Phylogeny of the Ampelocissus–Vitis clade in Vitaceae supports the New World origin of the grape genus. *Mol Phylogenet Evol* 95**,** 217-228. doi: [10.1016/j.ympev.2015.10.013](https://doi.org/10.1016/j.ympev.2015.10.013)

Lu, L.M., Mao, L.F., Yang, T., Ye, J.F., Liu, B., Li, H.L., Sun, M., Miller, J.T., Mathews, S., Hu, H.H., Niu, Y.T., Peng, D.X., Chen, Y.H., Smith, S.A., Min, C., Xiang, K.L., Le, C.T., Dang, V.C., Lu, A.M., Soltis, P.S., Soltis, D.E., Li, J.H., and Chen, Z.D. (2018). Evolutionary history of the angiosperm flora of China. *Nature* 554**,** 234-238. doi: 10.1038/nature25485

Luo, D., Yue, J.P., Sun, W.G., Xu, B., Li, Z.M., Comes, H.P., and Sun, H. (2016). Evolutionary history of the subnival flora of the Himalaya‐Hengduan Mountains: First insights from comparative phylogeography of four perennial herbs. *J Biogeogr* 43**,** 31-43. doi: [10.1111/jbi.12610](https://doi.org/10.1111/jbi.12610)

Ma, B.W., Hu, T.Y., Li, P., Yuan, Q.J., Lin, Z.S., Tu, Y.H., Li, J., Zhang, X.N., Wu, X.Y., Wang, X.J., Huang, L.Q., and Gao, W. (2017a). Phylogeographic and phylogenetic analysis for *Tripterygium* species delimitation. *Ecol Evol* 7**,** 8612-8623. doi: [10.1002/ece3.3344](https://doi.org/10.1002/ece3.3344)

Ma, Q., Du, Y.J., Chen, N., Zhang, L.Y., Li, J.H., and Fu, C.X. (2015). Phylogeography of *Davidia involucrata* (Davidiaceae) Inferred from cpDNA Haplotypes and nSSR Data. *Syst Bot* 40**,** 769-810. doi: [10.1600/036364415x689267](https://doi.org/10.1600/036364415x689267)

Ma, X.G., Sun, W.G., Zhu, W.D., and Sun, H. (2017b). Resolving the phylogenetic relationships and evolutionary history of the East Asian endemic genus *Rodgersia* (Saxifragaceae) using multilocus data. *Perspect Plant Ecol* 25**,** 20-28. doi: [10.1016/j.ympev.2011.04.022](https://doi.org/10.1016/j.ympev.2011.04.022)

Ma, X.G., Wang, Z.W., Tian, B., and Sun, H. (2019). Phylogeographic analyses of the east asian endemic genus *Prinsepia* and the role of the east asian monsoon system in shaping a north-south divergence pattern in China. *Front Genet* 10**,** 128. doi: [10.3389/fgene.2019.00128](https://doi.org/10.3389/fgene.2019.00128)

Niu, Y.T., Ye, J.F., Zhang, J.L., Wan, J.Z., Yang, T., Wei, X.X., Lu, L.M., Li, J.H., and Chen, Z.D. (2018). Long-distance dispersal or postglacial contraction? Insights into disjunction between Himalaya–Hengduan Mountains and Taiwan in a cold-adapted herbaceous genus, *Triplostegia*. *Ecol Evol* 8**,** 1131-1146. doi: [10.1002/ece3.3719](https://doi.org/10.1002/ece3.3719)

Qi, X.S., Chen, C., Comes, H.P., Sakaguchi, S., Liu, Y.H., Tanaka, N., Sakio, H., and Qiu, Y.X. (2012). Molecular data and ecological niche modelling reveal a highly dynamic evolutionary history of the East Asian Tertiary relict *Cercidiphyllum* (Cercidiphyllaceae). *New Phytol* 196**,** 617-630. doi: [10.1111/j.1469-8137.2012.04242.x](https://doi.org/10.1111/j.1469-8137.2012.04242.x)

Qi, X.S., Yuan, N., Comes, H.P., Sakaguchi, S., and Qiu, Y.X. (2014). A strong 'filter' effect of the East China Sea land bridge for East Asia's temperate plant species: inferences from molecular phylogeography and ecological niche modelling of *Platycrater arguta* (Hydrangeaceae). *BMC Evol Biol* 14**,** 14-41. doi: [10.1186/1471-2148-14-41](https://doi.org/10.1186/1471-2148-14-41)

Qiu, Y.X., Sun, Y., Zhang, X.P., Lee, J., Fu, C.X., and Comes, H.P. (2009). Molecular phylogeography of East Asian *Kirengeshoma* (Hydrangeaceae) in relation to Quaternary climate change and landbridge configurations. *New Phytol* 183**,** 480-495. doi: [10.1111/j.1469-8137.2009.02876.x](https://doi.org/10.1111/j.1469-8137.2009.02876.x)

Renner, S.S., and Zhang, L.B. (2004). Biogeography of the *Pistia* clade (araceae): Based on chloroplast and mitochondrial DNA sequences and bayesian divergence time inference. *Syst Biol* 53**,** 422-432. doi: [10.1080/10635150490445904](https://doi.org/10.1080/10635150490445904)

Schaefer, H., Heibl, C., and Renner, S.S. (2009). Gourds afloat: a dated phylogeny reveals an Asian origin of the gourd family (Cucurbitaceae) and numerous oversea dispersal events. *P Roy Soc B-Biol Sci* 276**,** 843-851. doi: [10.1098/rspb.2008.1447](https://doi.org/10.1098/rspb.2008.1447)

Schwery, O., Onstein, R.E., Bouchenak-Khelladi, Y., Xing, Y., Carter, R.J., and Linder, H.P. (2015). As old as the mountains: the radiations of the Ericaceae. *New Phytol* 207**,** 355-367. doi: [10.1111/nph.13234](https://doi.org/10.1111/nph.13234)

Setoguchi, H., Yukawa, T., Tokuoka, T., Momohara, A., Sogo, A., Takaso, T., and Peng, C.-I. (2006). Phylogeography of the genus *Cardiandra* based on genetic variation in cpDNA sequences. *J Plant R* 119**,** 401-405. doi: 10.1007/s10265-006-0283-1

Sophia, W.-P.H., and Jury, S.L. (2011). Phylogeny and divergence times inferred from rps16 sequence data analyses for *Tricyrtis* (Liliaceae), an endemic genus of north-east Asia. *AoB PLANTS* 2011, plr025. doi: [10.1093/aobpla/plr025](https://doi.org/10.1093/aobpla/plr025)

Sun, Q.H., Morales-Briones, D.F., Wang, H.X., Landis, J.B., Wen, J., and Wang, H.F. (2021). Phylogenomic analyses of the East Asian endemic *Abelia* (Caprifoliaceae) shed insights into the temporal and spatial diversification history with widespread hybridization. *Ann Bot-London* 129**,** 201-216. doi: [10.1093/aob/mcab139](https://doi.org/10.1093/aob/mcab139)

Sun, Y.X., Moore, M.J., Yue, L.L., Feng, T., Chu, H.J., Chen, S.T., Ji, Y.H., Wang, H.C., and Li, J.Q. (2014). Chloroplast phylogeography of the East Asian Arcto-Tertiary relict *Tetracentron sinense* (Trochodendraceae). *J Biogeogr*  41**,** 1721-1732. doi: [10.1111/jbi.12323](https://doi.org/10.1111/jbi.12323)

Tian, S., Lei, S.Q., Wan, H., Deng, L.L., Bo, L., Meng, Q.L., Soltis, D.E., Soltis, P.S., Fan, D.M., and Zhang, Z.Y. (2015). Repeated range expansions and inter-/postglacial recolonization routes of *Sargentodoxa cuneata* (Oliv.) Rehd. et Wils. (Lardizabalaceae) in subtropical China revealed by chloroplast phylogeography. *Mol Phylogenet Evol* 85**,** 238-246. doi: [10.1016/j.ympev.2015.02.016](https://doi.org/10.1016/j.ympev.2015.02.016)

Tu, T.Y., Volis, S., Dillon, M.O., Sun, H., and Wen, J. (2010). Dispersals of Hyoscyameae and Mandragoreae (Solanaceae) from the New World to Eurasia in the early Miocene and their biogeographic diversification within Eurasia. *Mol Phylogenet Evol* 57**,** 1226-1237. doi: [10.1016/j.ympev.2010.09.007](https://doi.org/10.1016/j.ympev.2010.09.007)

Valcárcel, V., Fiz-Palacios, O., and Wen, J. (2014). The origin of the early differentiation of *Ivies* (Hedera L.) and the radiation of the Asian Palmate group (Araliaceae). *Mol Phylogenet Evol* 70**,** 492-503. doi: [10.1016/j.ympev.2013.10.016](https://doi.org/10.1016/j.ympev.2013.10.016)

Wang, G.N., He, X.Y., Miehe, G., and Mao, K.S. (2014). Phylogeography of the Qinghai–Tibet Plateau endemic alpine herb *Pomatosace filicula* (Primulaceae). *J Syst Evol* 52**,** 289-302. doi: 10.1111/jse.12089

Wang, H.F., Landrein, S., Dong, W.P., Nie, Z.L., Kondo, K., Funamoto, T., Wen, J., and Zhou, S.L. 2015. Molecular phylogeny and biogeographic diversification of Linnaeoideae (Caprifoliaceae s. L.) disjunctly distributed in Eurasia, North America and Mexico. *PloS ONE* 10, e0116485. doi: [10.1371/journal.pone.0116485](https://doi.org/10.1371/journal.pone.0116485)

Wang, J.J., Yang, Y.P., Sun, H., Wen, J., Deng, T., Nie, Z.L., and Meng, Y. (2016). The biogeographic south-north divide of *Polygonatum* (Asparagaceae tribe Polygonateae) within Eastern Asia and its recent dispersals in the Northern Hemisphere. *PloS ONE* 11**,** e0166134. doi: [10.1371/journal.pone.0166134](https://doi.org/10.1371/journal.pone.0166134)

Wang, Y.H., Comes, H.P., Cao, Y.-N., Guo, R., Mao, Y.R., and Qiu, Y.X. (2017). Quaternary climate change drives allo-peripatric speciation and refugial divergence in the *Dysosma versipellis-pleiantha* complex from different forest types in China. *Sci Rep-UK* 7**,** 40261. doi: [10.1038/srep40261](https://doi.org/10.1038/srep40261)

Wang, Y.L., and Yan, G.Q. 2014. Molecular phylogeography and population genetic structure of *O. longilobus* and *O. taihangensis* (*Opisthopappus*) on the Taihang mountains. *PloS ONE* 9, e104773. doi: 10.1371/journal.pone.0104773

Wu, X.T., Ruhsam, M., Wen, Y., Thomas, P.I., Worth, J.R.P., Lin, X.Y., Wang, M.Q., Li, X.Y., Chen, L., Lamxay, V., Le Canh, N., and Coffman, G. (2019). The last primary forests of the Tertiary relict *Glyptostrobus pensilis* contain the highest genetic diversity. *Forestry* 93, 359-375. doi [10.1093/forestry/cpz063](https://doi.org/10.1093/forestry/cpz063)

Wu, Z.Y., Liu, J., Provan, J., Wang, H., Chen, C.J., Cadotte, M.W., Luo, Y.H., Amorim, B.S., Li, D.Z., and Milne, R.I. (2018). Testing Darwin's transoceanic dispersal hypothesis for the inland nettle family (Urticaceae). *Ecol Lett* 21**,** 1515-1529. doi: [10.1111/ele.13132](https://doi.org/10.1111/ele.13132)

Xiang, K.L., Zhao, L., Erst, A.S., Yu, S.X., Jabbour, F., and Wang, W. (2017). A molecular phylogeny of *Dichocarpum* (Ranunculaceae): Implications for eastern Asian biogeography. *Mol Phylogenet Evol* 107**,** 594-604. doi: [10.1016/j.ympev.2016.12.026](https://doi.org/10.1016/j.ympev.2016.12.026)

Xie, D.F., Li, M.J., Tan, J.B., Price, M., Xiao, Q.Y., Zhou, S.D., Yu, Y., and He, X.J. (2017). Phylogeography and genetic effects of habitat fragmentation on endemic *Urophysa* (Ranunculaceae) in Yungui Plateau and adjacent regions. *PloS ONE* 12**,** e0186378. doi: [10.1371/journal.pone.0186378](https://doi.org/10.1371/journal.pone.0186378)

Xie, P.W., Tu, T.Y., Razafimandimbison, S.G., Zhu, C.J., and Zhang, D.X. (2014). Phylogenetic position of *Guihaiothamnus* (Rubiaceae): Its evolutionary and ecological implications. *Mol Phylogenet Evol* 78**,** 375-385. doi: [10.1016/j.ympev.2014.05.022](https://doi.org/10.1016/j.ympev.2014.05.022)

Yang, L.Q., Hu, H.Y., Xie, C., Lai, S.P., Yang, M., He, X.J., and Zhou, S.D. (2017). Molecular phylogeny, biogeography and ecological niche modelling of *Cardiocrinum* (Liliaceae): insights into the evolutionary history of endemic genera distributed across the Sino-Japanese floristic region. *Ann Bot-London* 119**,** 59-72. doi: [10.1093/aob/mcw210](https://doi.org/10.1093/aob/mcw210)

Yu, W.B., Randle, C.P., Lu, L., Wang, H., Yang, J.B., Depamphilis, C.W., Corlett, R.T., and Li, D.Z. (2018). The hemiparasitic plant *Phtheirospermum* (orobanchaceae) is polyphyletic and contains cryptic species in the hengduan mountains of southwest China. *Front Plant Sci* 9**,** 142. doi: [10.3389/fpls.2018.00142](https://doi.org/10.3389/fpls.2018.00142)

Yuan, Q.J., Zhang, Z.-Y., Peng, H., and Ge, S. (2008). Chloroplast phylogeography of *Dipentodon* (Dipentodontaceae) in southwest China and northern Vietnam. *Mol Ecol* 17**,** 1054-1065. doi: [10.1111/j.1365-294X.2007.03628.x](https://doi.org/10.1111/j.1365-294x.2007.03628.x)

Yue, J.P., Sun, H., Baum, D.A., Li, J.H., Al-Shehbaz, I.A., and Ree, R. (2009). Molecular phylogeny of *Solms-laubachia* (Brassicaceae) s.l., based on multiple nuclear and plastid DNA sequences, and its biogeographic implications. *J Systemat Evol* 47**,** 402-415. doi: [10.1111/j.1759-6831.2009.00041.x](https://doi.org/10.1111/j.1759-6831.2009.00041.x)

Yue, X.K., Yue, J.P., Yang, L.E., Li, Z.M., and Sun, H. (2011). Systematics of the genus *Salweenia* (Leguminosae) from Southwest China with discovery of a second species. *TAXON* 60**,** 1366-1374. doi: [10.1002/tax.605012](https://doi.org/10.1002/tax.605012)

Zhang, J., Li, Z., Fritsch, P.W., Tian, H., Yang, A., and Yao, X. (2015a). Phylogeography and genetic structure of a Tertiary relict tree species, *Tapiscia sinensis* (Tapisciaceae): Implications for conservation. *Ann Bot-London* 116**,** 1-11. doi: [10.1093/aob/mcv112](https://doi.org/10.1093/aob/mcv112)

Zhang, J.W., Nie, Z.L., Wen, J., and Sun, H. (2011). Molecular phylogeny and biogeography of three closely related genera, *Soroseris*, *Stebbinsia*, and *Syncalathium* (Asteraceae, Cichorieae), endemic to the Tibetan Plateau, SW China. *Taxon* 60**,** 15-26. doi: [10.1002/TAX.601003](https://doi.org/10.1002/TAX.601003)

Zhang, M.L., Huang, J.F., Sanderson, S.C., Yan, P., Wu, Y.H., and Pan, B.R. (2015b). Molecular biogeography of tribe Thermopsideae (leguminosae): A madrean-tethyan disjunction pattern with an african origin of core genistoides. *Biomed Res Int* 864804. doi: [10.1155/2015/864804](https://doi.org/10.1155/2015/864804)

Zhang, X.Z., Zeng, C.X., Ma, P.F., Haevermans, T., Zhang, Y.X., Zhang, L.N., Guo, Z.H., and Li, D.Z. (2016a). Multi-locus plastid phylogenetic biogeography supports the Asian hypothesis of the temperate woody bamboos (Poaceae: Bambusoideae). *Mol Phylogenet Evol* 96**,** 118-129. doi: [10.1016/j.ympev.2015.11.025](https://doi.org/10.1016/j.ympev.2015.11.025)

Zhang, Y.H., Wang, I.J., Comes, H.P., Hua, P., and Qiu, Y.X. (2016b). Contributions of historical and contemporary geographic and environmental factors to phylogeographic structure in a Tertiary relict species, *Emmenopterys henryi* (Rubiaceae). *Sci Rep-UK* 6**,** 24041. doi: 10.1038/srep24041

Zhao, J.H., Zhou, P., Li, X.Q., Zhang, L.G., Jin, X.H., and Xiang, X.G. (2020). Temporal and spatial pattern of *Holcoglossum* schltr. (Orchidaceae), an East Asian endemic genus, based on nuclear and chloroplast genes. *Front Ecol Evol* 8**,** 245. doi: [10.3389/fevo.2020.00245](https://doi.org/10.3389/fevo.2020.00245)

Zhao, J.L., Xia, Y.M., Cannon, C.H., Kress, W.J., and Li, Q.J. (2016). Evolutionary diversification of alpine ginger reflects the early uplift of the Himalayan–Tibetan Plateau and rapid extrusion of Indochina. *Gondwana Res* 32**,** 232-241. doi: [10.1016/j.gr.2015.02.004](https://doi.org/10.1016/j.gr.2015.02.004)

Zhao, Y.F., Pan, B.R., and Zhang, M.L. (2019). Phylogeography and conservation genetics of the endangered *Tugarinovia mongolica* (Asteraceae) from Inner Mongolia, Northwest China. *PLoS ONE* 14**,** e0211696. doi: [10.1371/journal.pone.0211696](https://doi.org/10.1371/journal.pone.0211696)

Zhou, S.L., Renner, S.S., and Wen, J. (2006). Molecular phylogeny and intra- and intercontinental biogeography of Calycanthaceae. *Mol Phylogenet Evol* 39**,** 1-15. doi: [10.1016/j.ympev.2006.01.015](https://doi.org/10.1016/j.ympev.2006.01.015)

Zhou, Z., Hong, D.Y., Niu, Y., Li, G.D., Nie, Z.L., Wen, J., and Sun, H. (2013). Phylogenetic and biogeographic analyses of the Sino-Himalayan endemic genus *Cyananthus* (Campanulaceae) and implications for the evolution of its sexual system. *Mol Phylogenet Evol* 68**,** 482-497. doi: [10.1016/j.ympev.2013.04.027](https://doi.org/10.1016/j.ympev.2013.04.027)
